# Effect of HKUST-1 metal–organic framework in root and shoot systems, as well as seed germination

**DOI:** 10.1007/s11356-023-31728-6

**Published:** 2024-01-20

**Authors:** Sandra Loera-Serna, Hiram I. Beltrán, Mariana Mendoza-Sánchez, Juan Carlos Álvarez-Zeferino, Fernando Almanza, Fabián Fernández-Luqueño

**Affiliations:** 1 Universidad Autónoma Metropolitana Unidad Azcapotzalco, Av. San Pablo 420, Col. Nueva El Rosario, Alcaldía Azcapotzalco, C.P. 02128 Ciudad de Mexico, Mexico; 2 Sustainability of Natural Resources and Energy Program, CINVESTAV-Saltillo, Parque Industrial Saltillo-Ramos Arizpe, Av. Industrial Metalúrgica 1062, C.P. 25900 Ramos Arizpe, Saltillo, Coahuila Mexico

**Keywords:** Phytotoxicity, Seed germination, Root growth, HKUST-1, Shoot system, MOF

## Abstract

The seed germination, as well as root and shoot growth effect of HKUST-1 MOF, and its derived linear polymer ([Cu_2_(OH)(BTC)(H_2_O)]_*n*_·2*n*H_2_O) were herein examined. These effects were studied for seven higher plant species: sweet corn (*Zea mays* L.), black bean (*Phaseolus vulgaris* L*.*), tomato (*Solanum lycopersicum* L.), lettuce (*Lactuca sativa* L*.*), celosia *(Celosia argentea* L.), Aztec marigold* (Tagetes erecta* L.), and gypsophila (*Gypsophila paniculata* L.). The studied concentrations of MOFs were 10, 100, 500, or 1000 mg/L, enhancing the percentage of germination and growth of plants in most species. In general, the growth of the root is lower compared to the controls due to the capacity of the MOF to adsorb water and provide micronutrients such as C, O, and Cu, acting as a reserve for the plant. Shoot system growths are more pronounced with HKUST-1 compared with control, and linear polymer, due to the 3D structure adsorbs major water contents. It was found that all studied species are tolerant not only to Cu released from the material, but more evident to Cu structured in MOFs, and this occurs at high concentrations compared to many other systems. Finally, copper fixation was not present, studied by EDX mapping, banning the possibility of metallic phytotoxicity to the tested cultivars.

**Supplementary Information:**

The online version contains supplementary material available at 10.1007/s11356-023-31728-6.

## Introduction

Metal–organic frameworks (MOFs) or structured coordination polymers (COPs) are considered a new class of materials, whose boom has increased rapidly due to a large number of potential applications. These compounds develop 1D, 2D, or 3D structures, and are constituted by metal nodes and bridging organic ligands. Although it is expected that the toxicological properties of these materials are provided by (i) solely or merely because of the metal center, and (ii) the whole structure, nevertheless, this metal–ligand relation plays an important role in the mechanisms of release of the metallic atoms. Due to the physicochemical characteristics presented by MOFs (high surface area, tunable pore size, and particle morphology), these materials have been studied in applications such as catalysis, drug delivery, guest adsorption (molecular recognition), optical applications, sensor technologies, luminescence, and gas storage (Bon 2017; Burrows 2017; Loera-Serna et al. 2016).

One of the most studied MOFs is HKUST-1 (or Cu_3_(BTC)_2_, BTC: benzene-1,3,5-tricarboxylate); its main structural feature is a secondary building unit (SBU), called paddlewheel, containing a di-copper cluster with a copper–copper distance of 2.63 Å surrounded by eight oxygen atoms, from the carboxylate groups of the benzene-1,3,5-tricarboxylate (BTC) ligands. This motif binds to four coordination sites of each of the two copper ions, giving place to two Cu-coordinate water molecules placed in axial positions or also to other donor atom-containing molecules (Lewis bases). These paddle-wheel units form a face-centered crystal lattice with Fm-3m symmetry, with cell parameter and cell volume of 26.34 Å and 18,213 Å^3^, respectively, which possesses a three-dimensional channel system with a bimodal pore size distribution (Chui et al. 1999). This MOF could be synthesized at room temperature with a particle size in the order of a few nanometers (Loera-Serna et al. 2013). Due to these properties, HKUST-1 has been studied for several applications (Lin and Hsieh 2015, Loera-Serna et al. 2012, 2017, Yang and Zhang 2017); anyhow, this material presents low stability in aqueous solution since the characteristic 3D structure changed to [Cu_2_(OH)(BTC)(H_2_O)]_*n*_·2*n*H_2_O coordination polymer, which contains 5 × 7 Å dimension lozenge shaped 1D open channels along the crystallographic *a* axis (Chen et al. 2003). Therefore, in aqueous media, these two structures are certainly present depending on experimental conditions.

The non-toxic nanometric particle sizing achieved in these materials is of crucial interest for bioapplications such as the controlled release of therapeutic molecules or imaging (Chalati et al. 2011; Horcajada et al. 2010, 2006; Taylor et al. 2008; Xiao et al. 2009; Xie et al. 2007). To prepare large-scale uniform nanoparticulate MOF crystals for practical applications, several synthetic schemes have been developed, including methods: microwave-assisted (Zou et al. 2013), solvothermal (Wang et al. 2013), microemulsion (Rieter et al. 2006), sonochemical (Armstrong et al. 2017), and direct mixing (Li et al. 2009).

Currently, the application of MOF in agricultural and food processes started to appear but the main problem has been the inherent toxicity of MOFs (Ettlinger et al. 2022, Sajid and Ihsanullah 2020). Zhang et al. investigated the development and application of MOF to encapsulate ethylene and its release to stimulate ripening in climacteric fruits (Zhang et al. 2016). They found that MOF-ethylene treatment accelerated the ripening process of avocados and green banana fruits by reducing issue firmness and facilitating the ripening-related color change. In another work, Anstoetz et al. (2015) investigated the impact of the synthesized oxalate-phosphate-amine-MOF (OPA-MOF) on the growth, nutrient uptake, and grain yield of wheat (*Triticum aestivum* L.). They found that OPA-MOF has the potential to be used as novel enhanced efficiency N fertilizers. Lui et al. studied the effect of Fe-MOF and post-modified MOF with ETDA on growth, chlorophyll content, antioxidant activity, and iron uptake by *Phaseolus vulgaris* plant. They found that the enrichment effect of the Fe-MOF-EDTA component in nutrient solutions may be adopted as a strategy for improving the growth and quality parameters of *Phaseolus vulgaris* plants (Abdelhameed et al. 2019).

Because of all these statements, in this work, we studied the phytotoxicity of two copper MOFs (i) 3D HKUST-1 and (ii) [Cu_2_(OH)(BTC)(H_2_O)]_*n*_·2*n*H_2_O coordination polymer. The phytotoxicity of MOF materials was investigated by germination and means of both root as well as shoot systems elongation tests. The seeds of seven plants—sweet corn (*Zea mays* L.), black bean (*Phaseolus vulgaris* L*.*), tomato (*Solanum lycopersicum* L.), lettuce (*Lactuca sativa* L.), celosia *(Celosia argentea* L.*),* Aztec marigold (*Tagetes erecta* L.), and gypsophila (*Gypsophila paniculata* L.)—were investigated. The main objective of the present experiment was to test the hypothesis concerning if one of the most studied MOF (HKUST-1) and its derived linear polymer ([Cu_2_(OH)(BTC)(H_2_O)]_*n*_·2*n*H_2_O) could be used as high-efficient and environment-friendly amendments to alleviate the toxicity of copper to the plant species.

## Experimental procedure

### Materials

Benzene-1,3,5-tricarboxylic acid (BTC, 95%), copper nitrate (99.99%), anhydrous ethanol (99%), and sodium bicarbonate (> 99.7%), were purchased from Sigma-Aldrich. Deionized water and anhydrous ethanol were used as solvents. All the chemicals were used as received.

### HKUST-1 synthesis

The quantity of 2.38 mmol of BTC and 7.14 mmol of NaHCO_3_ was dissolved in 150 mL of deionized water. Then, a solution containing 3.57 mmol of copper nitrate trihydrate and 40 mL of ethanol was added dropwise; thus, HKUST-1 was prepared using the reported procedure (Loera-Serna et al. 2013). The synthetic mixture was stirred at room temperature for 12 h. The resulting HKUST-1 product was isolated by centrifugation and dried at 100 °C for 2 h.

### *[Cu*_*2*_*(OH)(BTC)(H*_*2*_*O)]*_*n*_*·2nH*_*2*_*O synthesis*

The HKUST-1 was mixed with water and treated at 100 °C and 15 psi for 40 min leading to the formation of [Cu_2_(OH)(BTC)(H_2_O)]_*n*_·2*n*H_2_O phase (Chen et al. 2003; Seo et al. 2009). The resulting blue product was isolated by centrifugation and dried at 100 °C for 2 h.

### Material characterization

Materials were characterized by X-ray diffraction (XRD), Fourier Transform infrared spectroscopy (FTIR), thermogravimetric analysis (TGA), and scanning electron microscopy (SEM). XRD patterns were collected on a Philips X’PERT PRO powder diffractometer coupled to a copper anode X-ray tube, to identify the crystallinity and characteristic phase of each sample. The Cu *Kα* radiation (45 kV, 40 mA, *k* = 1.5406 Å) was employed with a diffracted beam monochromator and a step size of 0.01° and a time per step of 0.9 s. The length of the XRD peaks at half-height determined the crystal size using the Debye–Scherrer equation. The FTIR spectra (4000–650 cm^−1^) were obtained with a resolution of 2 cm^−1^ at room temperature on a Bruker Tensor 27 spectrometer, fitted with a DTGS detector. The FTIR spectra were recorded through the ATR technique by diffuse reflectance. The TGA experiments were performed under an N_2_ atmosphere at a rate of 5 °C/min with a TGA Q500 (TA Instruments, USA). The samples were heated from room temperature to 500 °C to obtain the thermogram. Adsorption measurements were conducted using a BELSORP-max (BELL Japan Inc.) system at − 196 °C. Samples were degassed under dynamic conditions (extra-dry airflow) over 24 h at 100°C prior to N_2_ adsorption measurements. The BET specific surface areas were calculated from the N_2_ adsorption isotherms. The micrographs were acquired in a scanning electron microscope model Supra 55VP, with field emission cathode, in a high vacuum. The copper contents in the supernatants were detected by atomic absorption spectroscopy (Perkin Elmer, CP-OES Agilent series 5100).

### Seeds

Seeds of seven plant species—sweet corn (*Zea mays* L.), black bean (*Phaseolus vulgaris* L*.*), tomato (*Solanum lycopersicum* L.), lettuce (*Lactuca sativa* L.), celosia *(Celosia argentea* L.), Aztec marigold* (Tagetes erecta* L.), and gypsophila (*Gypsophila paniculata* L.)—were purchased from the Mexican Association of Seeds A.C. Three of the plant species (corn, tomato, lettuce) are among the 18 recommended species by EPA (2012) (EPA 2012) for the determination of ecological effects of pesticides and toxic substances. Other species are important in Mexico as food and for ornamental purposes; therefore, they were also tested for this means. Seeds were kept in a dry place in the dark at room temperature before use. The tracking of greenhouse temperature during the experiments was carried out.

### Germination

Seeds were immersed in a 5% sodium hypochlorite solution for 15 min to ensure surface sterility (EPA 2012); then, they were soaked in deionized water, after being washed several times with deionized water and kept in water for 15 min. Then, the seeds were removed and rewashed with deionized water. Seeds were transferred onto filter paper of 100 × 15 mm and placed in a Petri dish, with 10 seeds per dish and 1 cm or larger distance between each seed (Yang and Watts 2005). The quantity of 4 mL of MOF solution, of different concentrations among 10, 100, 500, or 1000 mg/L, was added to every seed species, and each experiment was carried out three times. This addition of MOF was only done once at the beginning of each experiment to keep the concentration of MOF constant. The dishes were covered and sealed with tape and placed in an incubator (Figure S1a). After 6 days in the dark under room temperature, more than 80% of the control seeds had germinated and developed at least 20 mm long roots. Then, the germination was halted, the seed germination rate was calculated, and the seedling root length was measured.

### Plant growth tests

The growth evaluation was carried out in polyethylene bags filled with garden soil. The bags were placed in glass pans with dimensions of 30 × 15 × 10 cm, that is, to group them by species (Figure S1b). It is worth mentioning that the seeds used in this test were not put in contact with any MOF prior to the growth test, that is, they are different seeds than those used for the germination tests, to keep the concentration of MOF constant in each experiment. Temperature and humidity were measured and recorded daily with USB Data loggers (RTH10). For each species, 100 g of the garden soil were weighed and poured into the polyethylene bag (100 g were considered enough material for germination means). Two seeds were embedded for each bag and each MOF concentration (10, 100, 500, or 1000 mg/L) with 5 replicates and one control. Solutions of 200 mL of each concentration of the Cu MOFs were made using water as solvent. The qualitative parameters of chlorosis, wilting, deformations, and necrosis of plant species were evaluated for 21 days. At the end of this time, the plant species were completely pulled out to measure the length of both roots and shoot systems with a ruler (precision of ± 0.1 cm) and count the number of leaves. Then, the obtained dry biomass was quantified and divided into shoot system biomass (leaves and stem) and root system biomass. The plant species were cut and rinsed with deionized water to remove soil residues. The roots were then introduced in paper bags and placed on a stove at 55 °C for 48 h; the same was done for the stem and leaves (shoot). The samples were weighed in an analytical balance (OHUS brand, with an accuracy of 0.0001 g), representing the dry biomass of the roots and the shoot systems (OECD Test No. 208 2006).

### Data analysis

An analysis of variance (ANOVA) of the results obtained in the previous tests was carried out. This analysis allowed correlating of the interaction between the factors (MOF type, the concentration of MOF, Cu, and vegetal species) and the response variables (length of shoot & root systems, as well as biomass weight) of all the tested populations and to see how they influenced the results (Walde 2014). The STATGRAPHICS Centurion XV software was used for this means, and statistical differences in interaction factors were also analyzed (Weinfurt 1995).

## Results and discussion

Herein, it has been surveyed the phytotoxic effect of two copper MOFs (i) 3D HKUST-1 and (ii) [Cu_2_(OH)(BTC)(H_2_O)]_*n*_·2*n*H_2_O coordination polymer. This has been assessed by seed germination as well as both root and shoot elongation tests of sweet corn, black bean, tomato, lettuce, celosia*,* Aztec marigold, and gypsophila, as will be detailed in the following sections.

### Characterization of Cu MOF

**Fig. 1:**
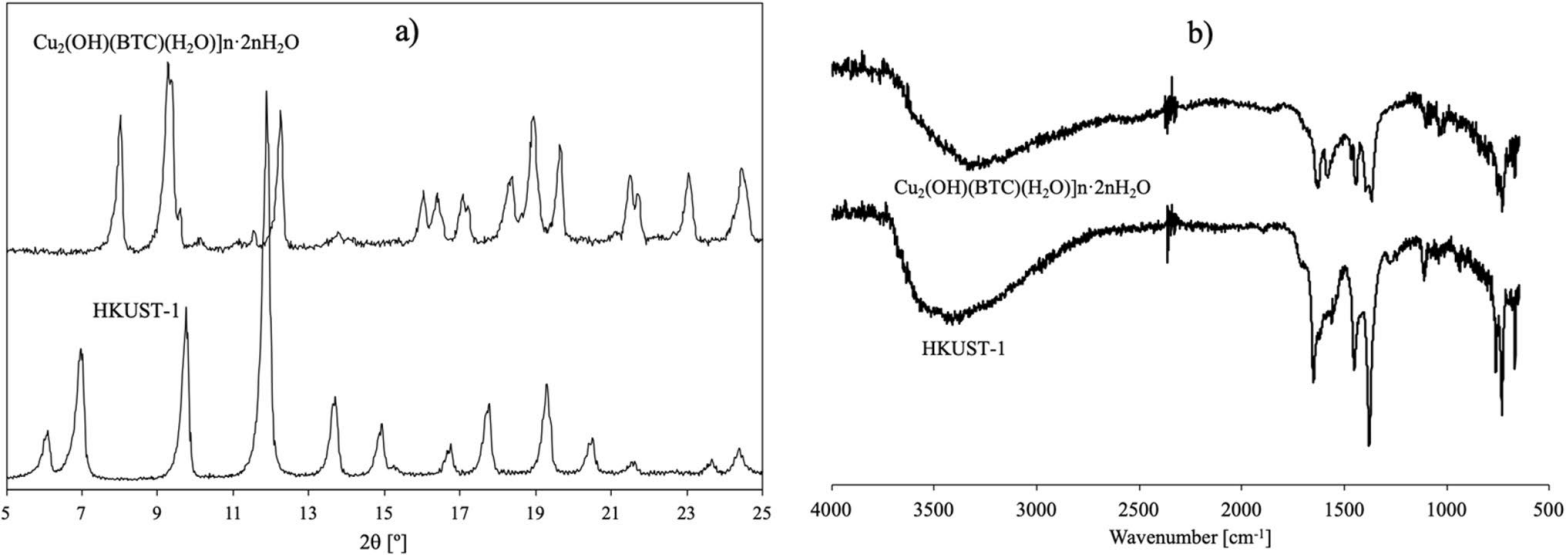
Characterization of [Cu_2_(OH)(BTC)(H_2_O)]_*n*_·2*n*H_2_O and HKUST-1. **a** XRD patterns and **b** FTIR

Synthesis of HKUST-1 was made by stirring at room temperature, Fig. 1a shows the obtained X ray diffraction. All the diffraction peaks of HKUST-1, as well as their shape and intensities, are quite coincident with those described elsewhere (Chui et al. 1999). For samples [Cu_2_(OH)(BTC)(H_2_O)]_*n*_·2*n*H_2_O, the diffraction patterns were different from that of HKUST-1 and indicated the formation of a coordination polymeric structure (Chen et al. 2003).

In the present case, there have been analyzed the FTIR spectra of the obtained materials (see Fig. 1b), where 3800–2700 broad bands are characteristic of water –OH groups with a high amount of hydrogen bonds, thus the amount of water into the pores resulted high. In the HKUST-1 sample, the formation of Cu_3_(BTC)_2_ structure is evident due to the presence of -C(OCu)OCu metallic esters in *ca.* 1704, 1648, 1591, 1548, 1448, 1419, and 1375 bands, indicating an *iso*bidentate coordination mode toward the two Cu sites in a very symmetric fashion. The metal centers are coordinated to the organic ligands and to water molecules that are adsorbed on the MOF (Loera-Serna et al. 2013). The FT-IR bands present in [Cu_2_(OH)(BTC)(H_2_O)]_*n*_·2*n*H_2_O clearly evidenced that the formation of HKUST-1 is not occurring. This is due to the presence of bands corresponding to -COOCu and also -COOH, and the absence of the band at 1703 cm^−1^ (always present in HKUST-1) both characteristic of the coordination polymer phase. In this phase, only two of the three carboxylates are present as Cu esters, and the remaining did not participate in the coordination, and thus remained free as -COOH functionalities (Loera-Serna et al. 2013).

Table 1 presents the physicochemical properties obtained by XRD, N_2_ adsorption, and TGA for [Cu_2_(OH)(BTC)(H_2_O)]_*n*_·2*n*H_2_O and HKUST-1. The surface area of HKUST-1 (1732.7 m^2^/g) and [Cu_2_(OH)(BTC)(H_2_O)]_*n*_·2*n*H_2_O (17.8 m^2^/g) confirmed the formation of 3D and non-porous structures, respectively. The cell parameter value of the HKUST-1 was smaller compared to that already reported (26.343 Å). This result suggested that the employed synthetic procedures provided materials with slightly compacted cells and thus of a bit smaller dimension. The crystal sizes of both samples were at the nanometric scale, 49.37 nm and 32.17 nm for HKUST-1 and [Cu_2_(OH)(BTC)(H_2_O)]_*n*_·2*n*H_2_O, respectively. While, the thermal stability was lower for [Cu_2_(OH)(BTC)(H_2_O)]_*n*_·2*n*H_2_O than the HKUST-1, due again to the different type, and dimensionality of the formed structure.

**Table 1:** Physicochemical properties of [Cu_2_(OH)(BTC)(H_2_O)]_*n*_·2*n*H_2_O and HKUST-1

	[Cu_2_(OH)(BTC)(H_2_O)]_*n*_·2*n*H_2_O	HKUST-1
Space group	P*n*	Fm-3m
BET specific surface area (m^2^/g)^a^	17.8	1732.7
Cell parameter (Å)^b^ Crystal size (nm)^c^ Thermal stability^d^	^e^ 32.17 275	26.31 49.37 323

^a^Determined by N_2_ adsorption, ^b^determined by XRD, ^c^determined by XRD using the Debye–Scherrer equation, ^d^determined by TGA, and ^e^not determined

**Fig. 2:**
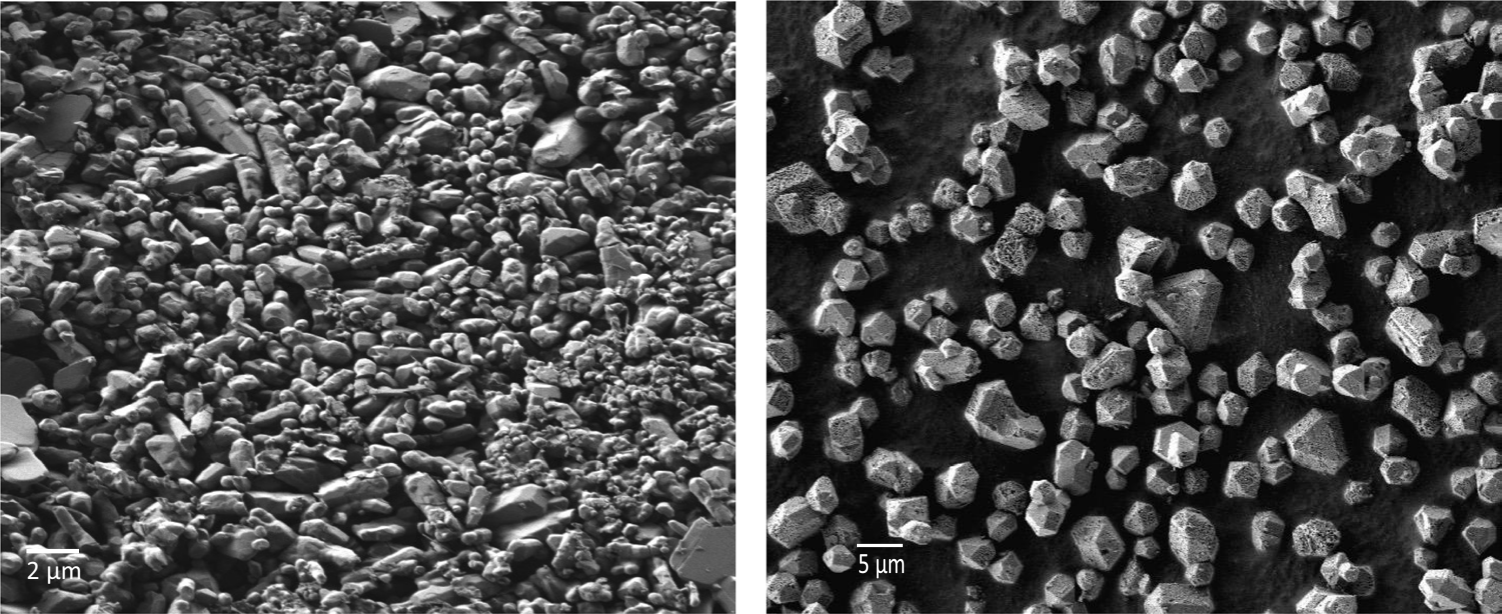
SEM images of [Cu_2_(OH)(BTC)(H_2_O)]_*n*_·2*n*H_2_O and HKUST-1 at 2.50 and 1.00 KX, respectively

Figure 2 shows the morphology of [Cu_2_(OH)(BTC)(H_2_O)]_*n*_·2*n*H_2_O and HKUST-1. Irregular particles were formed in the [Cu_2_(OH)(BTC)(H_2_O)]_*n*_·2*n*H_2_O sample with sizing between 0.3 and 3 μm. The octahedral crystals characteristic of HKUST-1 are shown in the SEM image of this sample, denoting homogenous sizes between 2 and 4 μm.

### Cu lixiviation in water

**Fig. 3:**
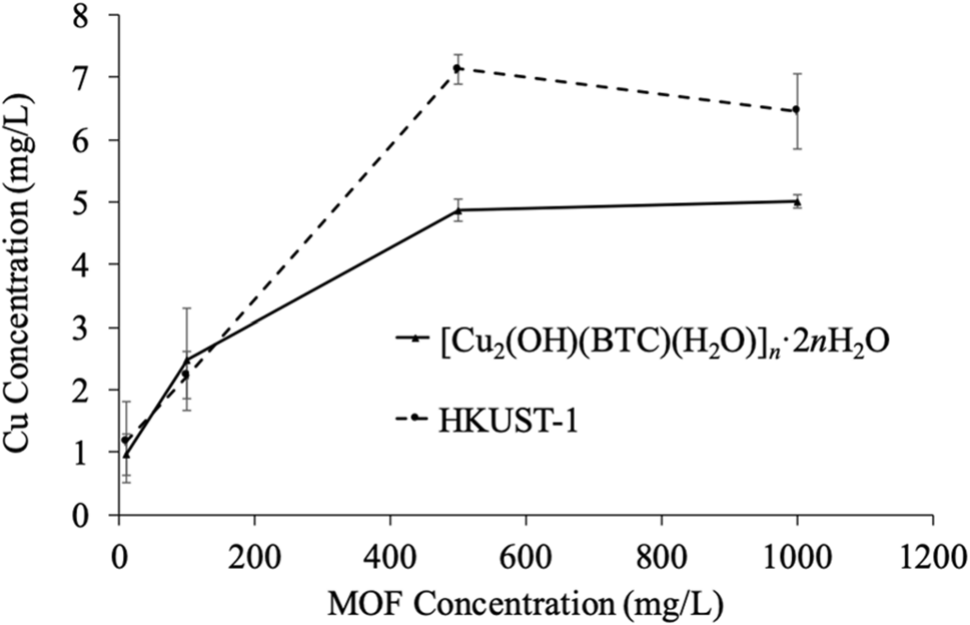
Cu dissolution from [Cu_2_(OH)(BTC)(H_2_O)]_*n*_·2*n*H_2_O and HKUST-1 (10, 100, 500, or 1000 mg/L) in deionized water. The values were given as the mean ± SD of triplicate samples

Total copper dissolution from HKUST-1 and [Cu_2_(OH)(BTC)(H_2_O)]_*n*_·2*n*H_2_O was determined in an aqueous solution after 4 days by atomic absorption spectroscopy. Monitoring was done every 24 h for a total period of 4 days, but since the copper concentration just slightly changed, as shown in Figure S2, the result after 4 days of treatment is presented in Fig. 3. The concentration of 500 and 1000 mg/L, less than 8 mg/L of copper, is released when the HKUST-1 was put in contact with deionized water, whereas for 10 and 100 mg/L concentrations, the release of copper was less than 3 mg/L.

The concentration of dissolved Cu increased rapidly with the increase of MOF concentration in both samples, but above 500 mg/L of MOF, the dissolved Cu tended to vary slightly with the variation in concentrations, being almost asymptotic. One important finding is that HKUST-1 released much more Cu than [Cu_2_(OH)(BTC)(H_2_O)]_*n*_·2*n*H_2_O sample at the same concentration. For instance, 1000 mg/L HKUST-1 released 6.46 mg/L of Cu, and only 5.01 mg/L of Cu were released from [Cu_2_(OH)(BTC)(H_2_O)]_*n*_·2*n*H_2_O. This difference, although small, is attributed to the different dimensionality, as well as the different amounts of copper in these two structures. Indeed, HKUST-1 has 3 equivalents of copper and 2 equivalents of BTC, and the non-porous structure ([Cu_2_(OH)(BTC)(H_2_O)]_*n*_·2*n*H_2_O) has 2 equivalents of copper for 1 equivalent of BTC. This evidence is a clear example of equilibrium since at lower concentrations the materials tend to displace to the solution as their ionic species. Meanwhile, at higher concentrations of the materials, the supersaturation state has been reached, minimizing the displacing of ionic species to the solution.

#### Cu MOF effect on early seedling growth

In order to evaluate the effect of copper MOF suspensions on the seed germination index (GI), and direct growth of the root and shoot systems of sweet corn, black bean, lettuce, tomato, celosia, Aztec marigold, and gypsophila, the concentrations of 0, 10, 100, 500, or 1000 mg/L of the [Cu_2_(OH)(BTC)(H_2_O)]_*n*_·2*n*H_2_O and HKUST-1 were employed in this investigation.

**Fig. 4:**
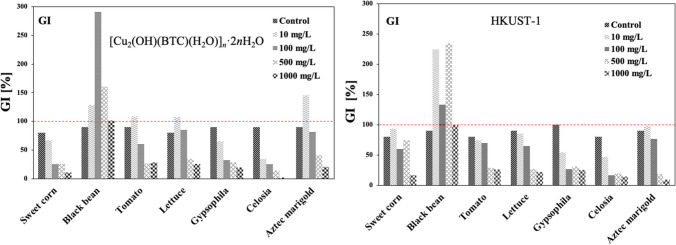
Histograms of germination index (GI) [%] of Cu MOF samples at different concentrations

Table 2 and Fig. 4 present the GI determined from the relation between the percentage of relative germination (PRG) and the relative growth of radicle (RGR), for which the following Eqs. (1–3) were applied:

**Table 2:** Germination index (GI) [%] of Cu MOF samples at different concentrations

	[Cu_2_(OH)(BTC)(H_2_O)]_*n*_·2*n*H_2_O [mg/L]					HKUST-1 [mg/L]				
	Control 1	10	100	500	1000	Control 2	10	100	500	1000
Sweet corn	80	67.45	24.98	25.63	10.73	80	93.58	60.30	74.40	16.69
Black bean	90	128.39	291.88	160.32	101.15	90	224.93	132.80	235.05	99.08
Tomato	90	108.72	59.86	26.15	27.87	80	73.97	69.67	28.44	25.52
Lettuce	80	107.24	85.40	34.16	25.64	90	85.12	64.46	27.00	21.56
Gypsophila	90	65.57	31.90	28.05	19.31	100	54.31	26.15	30.80	24.83
Celosia	90	34.11	25.47	14.21	1.79	80	46.63	16.79	19.21	13.95
Aztec marigold	90	145.23	81.04	40.37	20.33	90	97.88	75.91	18.56	9.29

1 $${\text{PRG}}=\frac{{\text{No}}.\mathrm{\;of\;germinated\;seeds\;in\;the\;extract}}{{\text{No}}.\mathrm{\;of\;germinated\;seeds\;in\;the\;control}}\cdot\;100$$

2 $${\text{RGR}}=\frac{\mathrm{Elongation\;of\;radicles\;in\;the\;extract}}{\mathrm{Elongation\;of\;radicles\;in\;the\;control}}\cdot\;100$$

3 $${\text{GI}}=\frac{{\text{PRG}}\times {\text{RGR}}}{100}$$

The controls 1 and 2 are related to percentages of viability for the seeds used in each germination test before the use of the linear polymer ([Cu_2_(OH)(BTC)(H_2_O)]_*n*_·2*n*H_2_O) or HKUST-1, respectively.

The effect of the linear polymer ([Cu_2_(OH)(BTC)(H_2_O)]_*n*_·2*n*H_2_O) for black bean*,* tomato, lettuce, and Aztec marigold is that the GI increases at 10 mg/L, obtaining values greater than 100% (control for these ranges between 90 and 80%), and favors germination; in these cases, it seems that the seeds are using the material as a complex micronutrient because previously, MOF is degrading in the presence of water, as seen in the “Cu lixiviation in water” section. Some species have low sensitivity to copper as a micronutrient like beans, grass, potatoes, and soybeans, and other cultivars reported medium sensitivity as barley, cauliflower, clover, corn, oats, sorghum, sugarbeet, and turnip (Gupta et al. 2008, Martens and Westermann 1991). However, for black beans at higher concentrations (100, 500, and 1000 mg/L), there are still GI values greater than 100%, and passing 100 mg/L started to decrease; nevertheless, these results could be attributed to the fact that the bean seeds used the linear material as a source of C, O, as well as Cu, where this type of plants have shown very good tolerances for even higher amounts of copper (Yurekli and Porgali 2006). For the rest of the treated samples and ranging from 100 to 1000 mg/L, there is a clear tendency to diminish germination while concentration increased. In particular, for sweet corn, gypsophila, and celosia, the trend is that GI decreased with increasing concentrations of linear material, but this occurred even at the lower dosage, with a minimum value of 1.70% for celosia at 1000 mg/L, being this cultivar the most affected at higher concentrations.

The data for HKUST-1 indicates that for sweet corn, tomato, lettuce, gypsophila, celosia, and Aztec marigold, the GI decreases with increasing concentration, being sweet corn and Aztec marigold, are a bit higher than control at the lower tested concentration (10 mg/L), and at higher concentrations, they also tend to diminish. Very contrasting results were found for black beans at concentrations of 10, 100, and 500 mg/L, where the GI exceeded the control and the 100%, reaching 225, 133, and 235%, respectively. It was observed that GI for sweet corn (74.4%), gypsophila (30.80%), and celosia (19.21%), at 500 mg/L, increased the GI compared with the concentrations of 100 and 1000 mg/L (60.30/16.69, 26.15/24.83, and 16.79/13.95%, respectively). These findings could be related to the concentration of released copper ions from the material (see Fig. 3), where HKUST-1 at 500 mg/L released 7.13 mg/L of copper to the solution, which is higher than that released by 1000 mg/L of HKUST-1, which just provided 6.46 mg/L of copper. This has been stated earlier, where the materials at lower concentrations released more ionic species to the solution, this indeed is a common thermodynamic effect related to the dissolution of salts and ionic supersaturation (Li and Demopoulos 2006). Indeed, the species that could be more adaptable to high concentrations of copper in solution were black bean > Aztec marigold > sweet corn > lettuce > tomato, and the less tolerant species resulted in gypsophila, and celosia.

By comparing the GI effect of both tested materials, the following trends were observed: values of GI for the linear polymer of the species black bean, tomato, lettuce, gypsophila, and Aztec marigold are greater than that of HKUST-1. For sweet corn and celosia, the HKUST-1 provoked higher GIs. Particularly, for sweet corn and Aztec marigold, the GI is higher for HKUST-1 than that for [Cu_2_(OH)(BTC)(H_2_O)]_*n*_·2*n*H_2_O; in these latter species, it seems to be important to maintain the copper in the structure of the MOF for germination purposes. When the species are in contact with [Cu_2_(OH)(BTC)(H_2_O)]_*n*_·2*n*H_2_O, a greater disposition of the Cu^2+^ ions is expected and, therefore, a greater contact of the metal with the medium. Interestingly, for black beans, the structure of the MOF/material is not a determining factor in the GI, since there is no trend in the results that determines a specific effect with the structure of the MOF.

### Effect of Cu MOF suspension on root growth

**Fig. 5:**
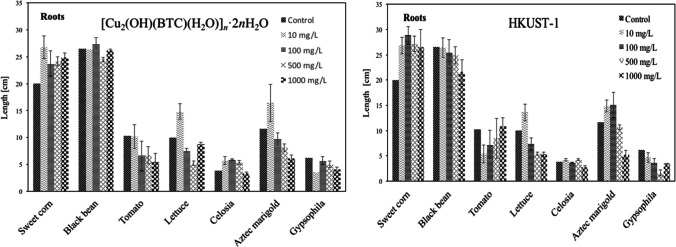
Root length of the seeds soaked and incubated in 10, 100, 500, or 1000 mg/L of [Cu_2_(OH)(BTC)(H_2_O)]_*n*_·2*n*H_2_O and HKUST-1 suspensions. The values were given as mean (standard deviation) of triplicate samples with 10 seeds each (*p* < 0.05)

The effects of [Cu_2_(OH)(BTC)(H_2_O)]_n_·2nH_2_O and HKUST-1 on the roots of all the tested cultivars are given in Fig. 5. Seedling root growth has been considered a rapid and widely used acute phytotoxicity test with several advantages: sensitivity, simplicity, low cost, and suitability for unstable chemicals or samples (Lin and Xing 2007, Munzuroglu and Geckil 2002, Wang et al. 2001). It is worth noting that just a few studies have been developed using metal–organic frameworks and related materials in phytotoxicity tests (Dandan 2022; Guan et al. 2021; Marqus et al. 2021; Zhang et al. 2021).

#### [Cu_2_(OH)(BTC)(H_2_O)]_n_·2nH_2_O effect

The results obtained herein indicate that [Cu_2_(OH)(BTC)(H_2_O)]_n_·2nH_2_O suspension had no obvious phytotoxicity on black beans, promoting the root growth of sweet corn, and celosia. For lettuce, tomato, Aztec marigold, and gypsophila the Cu linear MOF promoted the root growth at low concentrations (10 mg/L), and decreased at concentrations higher than 100 mg/L. The minimum growth on root length was observed for tomato and Aztec marigold with 47.38 and 47.41%, respectively at 1000 mg/L. In particular, optimum growth concentrations were found depending on each species, and the trend is as follows: the first resulted from sweet corn with 33.83% at 10 mg/L; black bean with 3.39% at 100 mg/L; lettuce with 48.33% at 10 mg/L; followed by celosia with 55.26% at 100 mg/L; and Aztec marigold with 41.67% at 10 mg/L. Comparatively, where only diminishment was assessed, with − 0.78% for tomatoes at 10 mg/L, which lies in the experimental error, and gypsophila, with − 8.06% at 100 mg/mL, also these being the optimum in their own trends, meaning the maximum growth once they interact with the linear coordination polymer.

#### HKUST-1 effect

For HKUST-1, the root growth had no obvious phytotoxicity on celosia, the same result was observed for the linear polymer. For sweet corn compared with the control, an increase in root length was observed for all studied concentrations. Meanwhile, for lettuce and Aztec marigold, an increase has been observed at low concentrations (10 mg/L), that later decreased at higher concentrations, compared with the control. For black beans and gypsophila, a continuous decrease is observed with the increase in concentration, again, compared with the control. In the case of tomato, at low concentrations (10 mg/L), the root length decreased and increased as the concentration of the MOF increased, reaching the value of the control at 1000 mg/L.

#### Comparative performance of [Cu_2_(OH)(BTC)(H_2_O)]_n_·2nH_2_O vs HKUST-1

The comparison of the values of root length of tested cultivars for the two materials did not show significant differences, all tested species have a similar root growth when compared to each other, and only the discrepancies described above were observed. The minimum growth is observed for celosia, but it is like that obtained for the control. The maximum root length assessed was for sweet corn in both cases; this means that the two materials were efficient in providing micronutrients (MOF elements as Cu, C, and O) in this type of plant (Gupta et al. 2008). Opposing, when Cu is directly added to the cultivars, diminishment of plant height with increasing Cu doses, causing toxic effects in maize plants with losses in growth and yield even at low concentrations of 3, 6, 9, and 12 mg/L (Mahmood et al. 2005), since it has been found that higher doses of Cu^2+^ (100, 200, 300, 400, 500, or 600 mg/L) caused the necrosis of shoot (Barbosa et al. 2013).

### Effect of Cu MOF suspension on shoot growth

**Fig. 6:**
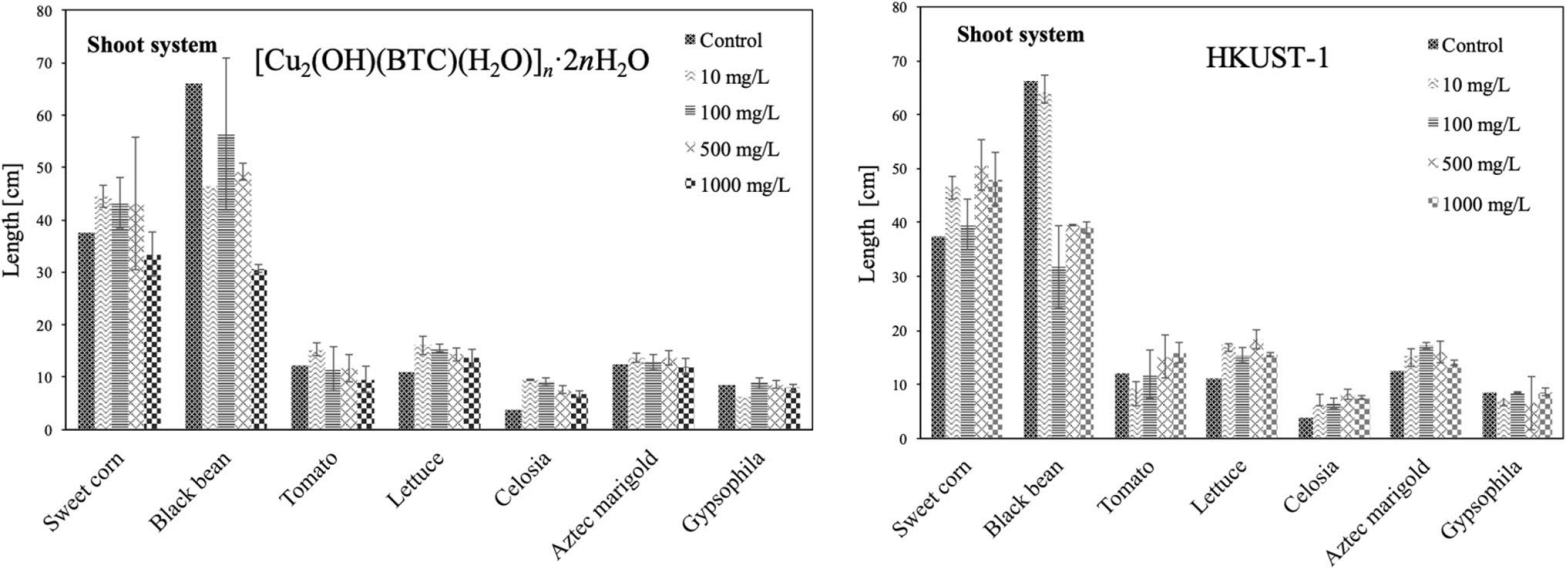
Shoot system of the seeds soaked and incubated in 10, 100, 500, or 1000 mg/L of [Cu_2_(OH)(BTC)(H_2_O)]_*n*_·2*n*H_2_O and HKUST-1 suspensions. The values were given as mean (standard deviation) of triplicate samples with 10 seeds each (*p* < 0.05)

The length of the shoot system is given in Fig. 6, for each species and for both tested materials, and the particular results will be detailed as follows.

#### [Cu_2_(OH)(BTC)(H_2_O)]_n_·2nH_2_O effect

Aztec marigold and gypsophila did not show significant differences (*p* < 0.005) from the controls (DI-water). The growth of the other species resulted higher than the control except for black beans, which clearly diminished. As has been evidenced for the roots, at 10 mg/L, there is a slight increase in length for sweet corn, tomato, lettuce, and celosia, and then subtly decreased at higher concentrations.

#### HKUST-1 effect

In the case of gypsophila did not show significant differences (*p* < 0.005) from the controls (DI-water). The other species, including Aztec marigold, growth more than the control except for the black beans which clearly diminished, very similar to the results provided by the linear polymer. As evidenced for the roots, at 10 mg/L, there is a slight increase in length for all species, but in this case of HKUST-1, varying behaviors were encountered. Only tomatoes did not enhance length at lower but at higher concentrations. Each species has its own optimum HKUST-1 concentrations, black bean at 10 mg/L, Aztec marigold and gypsophila at 100 mg/L, sweet corn, lettuce, and celosia are at 500 mg/L, and finally tomato at 1000 mg/L.

#### Comparative performance of [Cu_2_(OH)(BTC)(H_2_O)]_n_·2nH_2_O vs HKUST-1

The tendency in the results is the same for both Cu MOF. However, greater growth of the shoot system is observed when the plants are in contact with HKUST-1, for all tested species at 1000 mg/L. At lower concentrations, variations occurred, but most of the species have greater growth in the length of the shoot system with the HKUST-1. This result could be attributed to the use of MOF as a micronutrient; additionally, it is known that the 3D MOFs are capable of attaching/occluding different compounds/ions/species that could be gradually released (Loera-Serna et al. 2019, 2017, 2016), which would additionally function as a reservoir of water and other compounds able to promote the growth of the shoot system (Fu et al. 2023). Once the MOF is destroyed or disassembled, it releases the copper ions, as would the linear MOF, but this occurs only when the MOF is continuously exposed to an aqueous medium (Singh et al. 2016). Due to this, the MOF provides the plant with a higher amount of dosed water, C, O, and Cu; therefore, the roots did not need to be bigger for the development of the plant. In addition, the amount of final copper is not so high to generate an evident toxic effect, as found in other works (Barbosa et al. 2013; Mahmood et al. 2005). Recent studies have shown that copper from MOF could even be an antifungal agent due to the enzymatic activities of cellulases and amylases in plants (Abdelhameed et al. 2019).

### Effect of MOFs on the dry biomass content

**Fig. 7:**
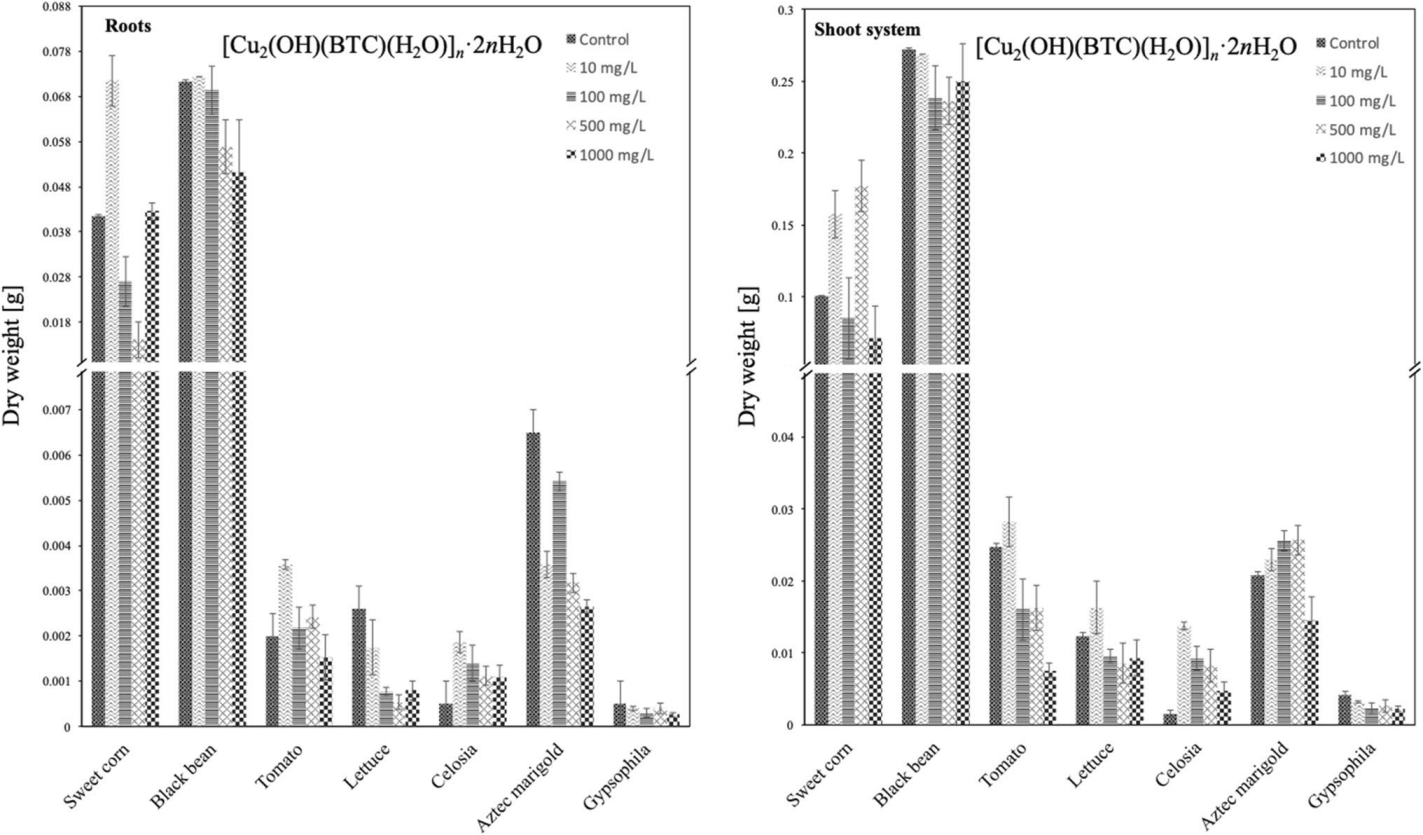
DW [g] of the roots and shoot system of each species in contact with [Cu_2_(OH)(BTC)(H_2_O)]_*n*_·2*n*H_2_O suspension at 10, 100, 500, or 1000 mg/L

**Fig. 8:**
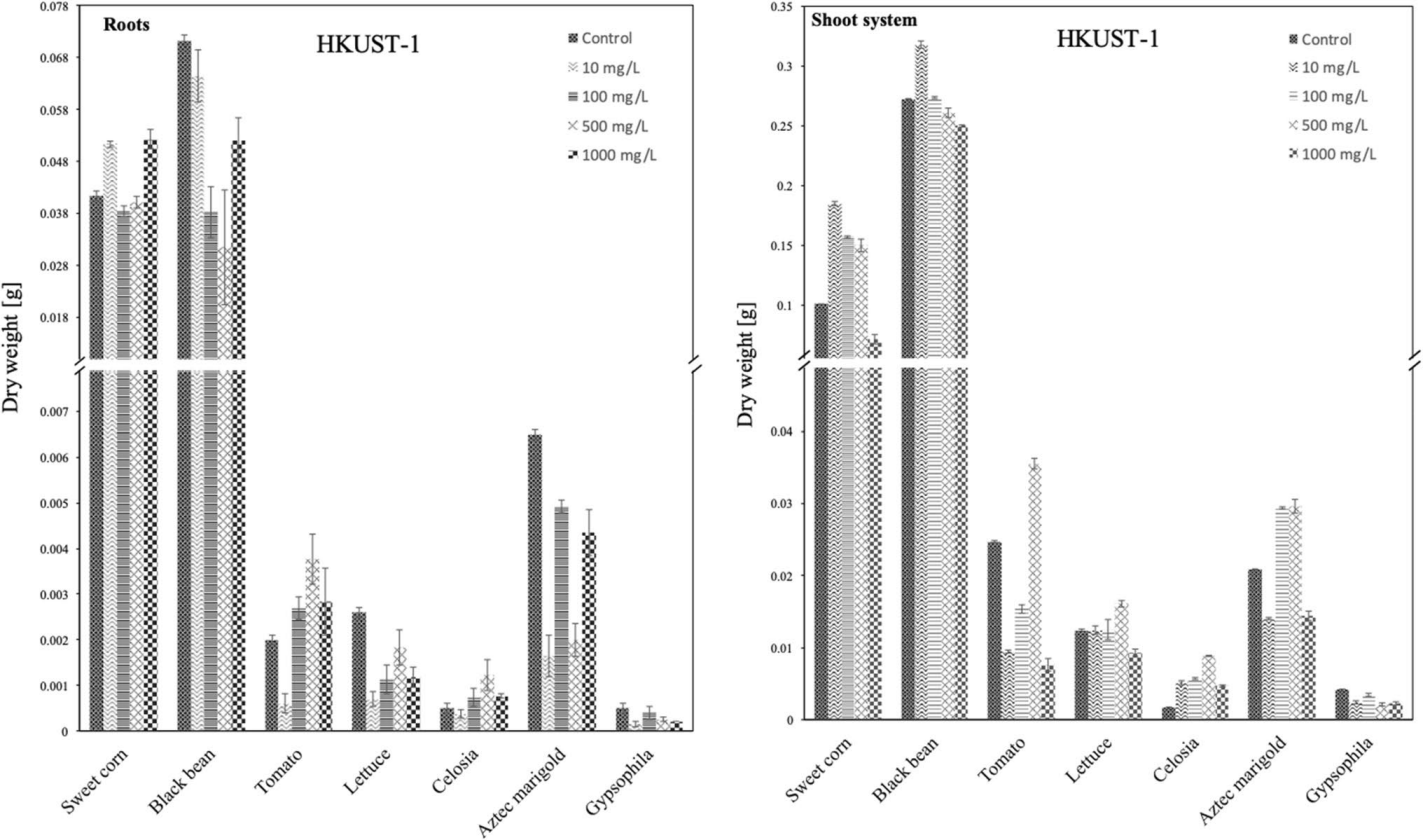
DW [g] of the roots and shoot system of each species in contact with HKUST-1 suspension at 10, 100, 500, or 1000 mg/L

Since the plants have high water composition and the water level in plants will depend on the amount of water in their environment (which is very difficult to control), using dry biomass as a measure of plant growth tends to be a more reliable determination. Therefore, dry biomass or dry weight (DW) measurements were determined after drying the material reaching a constant weight, being this a degree of the amount of protoplasm or dry matter. The results of DW are shown in Figs. 7 and 8 for linear polymer and HKUST-1 respectively. One general tendency is observed for sweet corn and black bean being the cultivars that presented the higher amounts of protoplasm or dry matter, in both root and shoot systems.

#### DW in roots caused by [Cu_2_(OH)(BTC)(H_2_O)]_n_·2nH_2_O

For the species of sweet corn, black bean, tomato, lettuce, gypsophila, and celosia, the optimum DW was found at concentrations of 10 mg/L, and from these, the only two that do not overpass control were lettuce and gypsophila. For the rest of the species, the dry weight decreased as the concentration of the material increased, with the exception of sweet corn, tomato, and celosia, which presented different DW modulations as a function of polymer concentration, in comparison to the control. At concentrations of 100 mg/L DW is lower compared to the control for all species except for Aztec marigold. Particularly, for celosia, the DW is higher compared to the control at all tested concentrations.

#### DW in shoot caused by [Cu_2_(OH)(BTC)(H_2_O)]_n_·2nH_2_O

DW optimum was found at 10 mg/L for black bean, tomato, lettuce, celosia, and gypsophila, being only black bean and gypsophila lower than the control. Optimum DW values for sweet corn and Aztec marigold were found at 500 mg/L. Again, celosia enhanced DW compared to the control at all tested concentrations, this case for both shoot and root systems. Aztec marigold also evidenced an enhanced DW, and only for the concentration of 1000 mg/L it becomes lower.

#### DW in roots caused by HKUST-1

This series evidenced varying and oscillating tendencies with intricate behavior related to DW and material dosage. The optimum DW was found at 1000 mg/L for sweet corn, at 500 mg/L for tomato, lettuce, and celosia, at 100 mg/L for Aztec marigold and gypsophila, meanwhile at 10 mg/L it is the black bean. The cultivars that developed higher amounts of DW in comparison to control were sweet corn, tomato, and celosia.

#### DW in shoots caused by HKUST-1

In all cases, there were found optimum DW tendencies, since optimum concentrations were at 10 mg/L of sweet corn and black bean, at 100 mg/L of only gypsophila, and for the rest at 500 mg/L. Herein is worth mentioning that all cultivars developed higher amounts of DW in comparison to the control, except for gypsophila.

#### Comparative performance of [Cu_2_(OH)(BTC)(H_2_O)]_n_·2nH_2_O vs HKUST-1

In general, the linear polymer presented optimum DW at lower concentration values for the root system, perhaps due to the higher availability of micronutrients in this open structure. Meanwhile, oscillating behaviors were found for HKUST-1 and at higher concentrations to achieve optimum DW values. These evident disparities arise from the differences in structure among both materials, being the most structured (HKUST-1), the most tolerated at higher concentrations both in roots and shoot system for the majority of cultivars.

In general, for the shoot system, DW optimum was found at 10 mg/L for almost all species, again due to the easy availability of micronutrients for the linear polymer. In comparison, optimum DW values were found for the most structured material in almost all cases at higher concentrations, again evidencing more tolerance of copper concentration for the structured material.

### Copper included in sweet corn species

The assay of inclusion of copper was only assessed for sweet corn, as representative of the fixation of this metal into the cultivar. It is known that Cu is needed by corn plants in small amounts, playing an active role in plant metabolism, measured as relative water content, chlorophyll content and fluorescence, photosynthesis as well as yield parameters (Liu et al. 2001; Mahmood et al. 2005; Tanyolac et al. 2007; Xin et al. 2022). In this line, as chosen species to analyze copper contents and due to the ability of this synergistic effect between sweet corn and copper, the presence of this metal in the studied species was determined through an EDX study (see Supporting Information File). The analysis was performed fivefold in both the root and the shoot systems, and Table 3 shows the results obtained from the average of each measure.

**Table 3:** Elemental analyses (%) through EDX for sweet corn species exposed to Cu MOFs materials

	Control		[Cu_2_(OH)(BTC)(H_2_O)]_*n*_·2*n*H_2_O		HKUST-1	
	Root	Shoot system	Root	Shoot system	Root	Shoot system
C	58.33 ± 1.18	57.66 ± 3.87	51.83 ± 0.87	54.40 ± 0.80	54.97 ± 1.22	57.72 ± 4.34
O	35.55 ± 0.83	36.13 ± 2.93	41.12 ± 0.53	38.44 ± 0.50	31.24 ± 0.59	36.28 ± 2.91
Na	0.27 ± 0.01	^a^	0.50 ± 0.03	^a^	0.23 ± 0.03	^a^
Mg	0.38 ± 0.05	0.17 ± 0.03	0.38 ± 0.04	0.31 ± 0.02	0.24 ± 0.05	0.21 ± 0.02
Al	0.53 ± 0.09	^a^	0.70 ± 0.02	^a^	0.34 ± 0.10	^a^
Si	1.23 ± 0.18	0.50 ± 0.33	1.57 ± 0.14	0.57 ± 0.04	0.40 ± 0.06	1.58 ± 0.70
P	0.36 ± 0.10	0.57 ± 0.10	0.26 ± 0.03	0.50 ± 0.03	0.31 ± 0.05	0.52 ± 0.11
S	0.45 ± 0.06	0.16 ± 0.05	0.55 ± 0.05	0.19 ± 0.02	0.23 ± 0.09	0.31 ± 0.02
Cl	0.18 ± 0.06	0.53 ± 0.08	0.18 ± 0.01	0.78 ± 0.14	0.32 ± 0.15	0.26 ± 0.09
K	1.61 ± 0.25	3.74 ± 0.51	1.70 ± 0.07	4.29 ± 0.58	0.62 ± 0.08	2.61 ± 0.52
Ca	0.83 ± 0.09	0.54 ± 0.11	0.96 ± 0.04	0.53 ± 0.07	0.60 ± 0.08	0.50 ± 0.02
Fe	0.29 ± 0.02	^a^	0.36 ± 0.03	^a^	^a^	^a^
Cu	^a,b^	^a,b^	^a,b^	^a,b^	^a,b^	^a,b^

^a^Not detected by EDX. ^b^ See Supporting Information File for evidence of the SEM mapping & EDX Spectrum, not detecting Cu contents

As expected, it is observed that the roots and the shoots have high concentrations of oxygen and carbon due to their organic matter nature. The presence of Na, Mg, Al, Si, P, S, Cl, K, Ca, and Fe was also observed. The absence of copper in the plants is corroborated in all the tested samples, hence probably present at lower percentages, since S and Mg were detected at 0.16 ± 0.05 and 0.17 ± 0.03, which were the lower percentages detected and present. This result indicates that despite using high concentrations (1000 mg/L) of copper into MOFs, this metal was not incorporated in any part of the plant, as far as we could determine, and at least for this species. Therefore, it could be that the MOF materials only worked as water and micronutrients reservoir, modifying the growth of the species, but not presenting phytotoxic effects, or copper overdosage, or copper overfixation.

### MOFs toxicity, cultivar/plants effects

In order to track the effect of HKUST-1 and its 1D linear coordination polymer toward certain cultivars’ properties, a comparison of the present results with already reported surveys on this topic resulted convenient. Two very interesting compendiums were found aimed at related research topics, one that develops a toxicity ranking of MOFs (Ettlinger et al. 2022) and the other aimed at the effects of adsorption/degradation of agrochemicals by MOFs and MOF Composites (Rojas et al. 2022). Nevertheless, for the current means, we have found a very related revision, named “metal–organic framework as an emerging material: a novel plant growth stimulant” (Chauhan et al. 2022), where the authors have stated that MOFs are considered to be smart delivery systems, mainly due to their slow delivery capacities of micronutrients within a limited period; therefore, as the authors stipulated, there is no need for monitoring for larger periods. Another interesting statement also found therein is that a smart delivery system is able to provide nutrients to all parts of plant/cultivar, roots, shoots, or fruits, and this occurred in short periods, banning the high loading of fertilizers or nutrients, and therefore minimizing plant damage/toxicity. Nevertheless, we have found that concentration varying is still needed to ascertain these observations since our results and some other results indicated oxidative stress appearance at higher concentrations. Because of these findings, we assembled Table 4 for the particular effect, that MOFs and derived materials have developed over reported cultivars properties, e.g., growing/yield of roots, shoots, or fruits. It was very interesting to note that the observed effects were very similar to those found in the current research.

**Table 4:** Effects of MOFs and related materials toward cultivars

Type of MOF	Cultivar/species	Effects	Ref
MOF-199 (HKUST-1)	Pea plants (*Pisum sativum L.*)	Accelerated germination of pea seeds, total germination rates unchanged. Inhibited seedling growth at high concentrations [1000 mg·L^−1^]. Net photosynthetic rate increased, total photosynthesis capability decreased. Acceptor side damage of photosystem II evidenced by chlorophyll fluorescence	(Guan et al. 2021)
MiZax-3@ZIF-8 (MiZIF)	Capsicum (*Capsicum annum*) crops	Enhanced fresh weight of tomato and pearl millet seedlings, MiZIF effectively released bioactive MiZax-3, promoting plant growth at early seedling stage	(Aguliar Perez et al. 2023)
OPA-MOF	Not tested on plants yet	New type of fertilizer, where microbial-assisted structural breakdown slowly releases the plant nutrient elements P, N, and Fe incorporated in the framework	(Anstoetz et al. 2016)
Fe-MOF	Not tested on plants yet	Nutrient (N, P, and Fe) release behaviors. Better release performance when placed in soil than when placed in water, and both compounds lasted longer than 100 days	(Wu et al. 2019)
OPA-MOF	Bread wheat (Triticum aestivum L.)	Potential as enhanced efficiency N fertilizer, but as P-bioavailability provider was insufficient to meet plant demands	(Anstoetz et al. 2015)

MiZax-3: synthetic mimetic of zaxinone (carotenoid-derived regulatory metabolite that promotes plant growth). OPA: oxalate-phosphate-amine

## Conclusions

This study surveyed the potential effects of [Cu_2_(OH)(BTC)(H_2_O)]_*n*_·2*n*H_2_O and HKUST-1 on copper metal tolerant plants. Cu MOFs promoted the growth of seven plant species: sweet corn (*Zea mays* L.), black bean (*Phaseolus vulgaris* L*.*), tomato (*Solanum lycopersicum* L.), lettuce (*Lactuca sativa* L*.*), celosia *(Celosia argentea* L.), Aztec marigold (*Tagetes erecta* L.), and gypsophila (*Gypsophila paniculata* L.). At low concentrations (< 100 mg/L), linear MOF acted as a reservoir of nutrients due to its easy availability according to its open structure, enhancing the percentage of germination and growth of plants in most species. In general, the growth of the root is lower compared to the controls due to the capacity of the MOF to adsorb water and provide micronutrients such as C, O, and Cu, acting as a reserve for the plant. The shoot system growths are more pronounced with HKUST-1 compared with control, and [Cu_2_(OH)(BTC)(H_2_O)]n·2nH_2_O and HKUST-1 due to the 3D structure adsorbs major water contents. Also, it has been found that both, HKUST-1 and linear polymer, deliver micronutrients in a particular higher dosage that the plants need for good development. Therefore, it could be stipulated that all studied species are tolerant not only to soluble Cu (released from the same material) but more evident to Cu structured as these framework materials, and this occurs at high concentrations compared to many other systems. Finally, it is worth mentioning that no matter of the high concentration employed for this means, copper fixation was not present, evidenced by EDX mapping; therefore, the possibility of metallic phytotoxicity to the tested cultivars has been banned with the use of [Cu_2_(OH)(BTC)(H_2_O)]n·2nH_2_O and HKUST-1.

### Supplementary Information

Below is the link to the electronic supplementary material.

Supplementary file1 (DOCX 3689 KB)

## Data Availability

All data supporting the findings of this study are included within the article (and any supplementary files).
