# Supplementary material for: Effect of HKUST-1 metal–organic framework in root and shoot systems, as well as seed germination
Source: Environ Sci Pollut Res Int. 2024 Jan 20;31(9):13270–83. doi: 10.1007/s11356-023-31728-6 (PMC10881711; doi:10.1007/s11356-023-31728-6)
Supplement: Supplementary file 1 — Supplementary file1 (DOCX 3689 KB) [file 11356_2023_31728_MOESM1_ESM.docx]

**SUPPORTING INFORMATION**

**Effect of HKUST-1 metal-organic framework in root & shoot systems, as well as seed germination**

*Sandra Loera-Serna.^1,*^, Mariana Mendoza-Sánchez^1^, Juan Carlos Álvarez-Zeferino,^1^ Fernando Almanza^1^, Hiram I. Beltrán,^1,*^ and Fabián Fernández-Luqueño^2^*

^1^ Universidad Autónoma Metropolitana Azcapotzalco, Av. San Pablo 420, Col. Nueva El Rosario, Azcapotzalco, Ciudad de México, 02128, México.

^2^ Sustainability of Natural Resources and Energy Program, CINVESTAV-Saltillo, Av. Industrial Metalúrgica 1062, Parque Industrial Saltillo-Ramos Arizpe, Ramos Arizpe Saltillo, Coahuila C.P. 25900, Mexico.

*E-mail: [sls@azc.uam.mx](mailto:sls@azc.uam.mx)

| 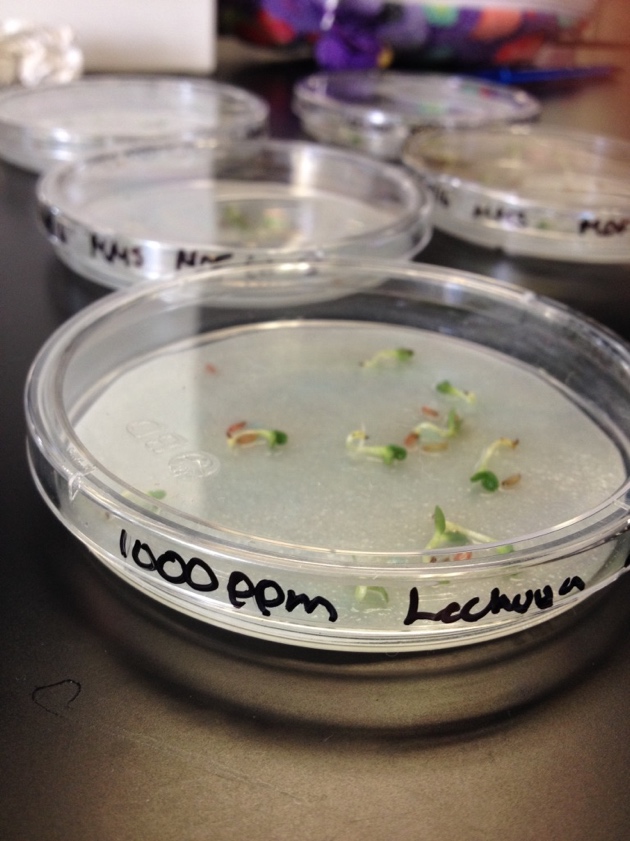 | 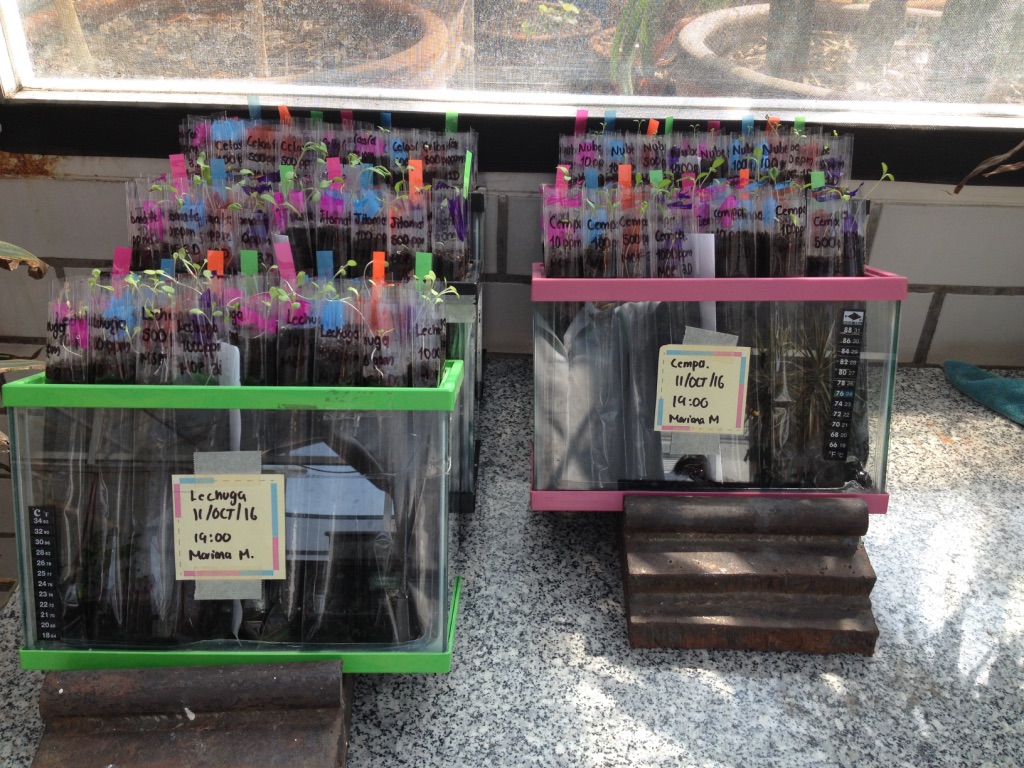 |
| --- | --- |
| **a)** | **b)** |
| **Figure S1**. a) Germination experiment of seeds and b) first instar growth test for the seven tested species. | |


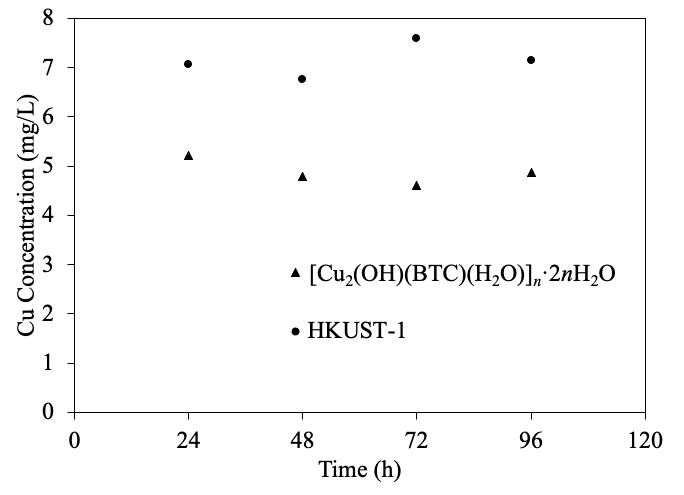


**Figure S2.** Cu dissolution from [Cu_2_(OH)(BTC)(H_2_O)]*_n_*·2*n*H_2_O and HKUST-1 at 500 mg/L in deionized water as a function of time.

**SEM and EDX of samples**

1. ***Control sample***
   1. Element composition data and mapping image for the root of sweet corn.

| Element composition | | | Mapping image |
| --- | --- | --- | --- |
| SEM | EDX data | EDX spectrum | C O  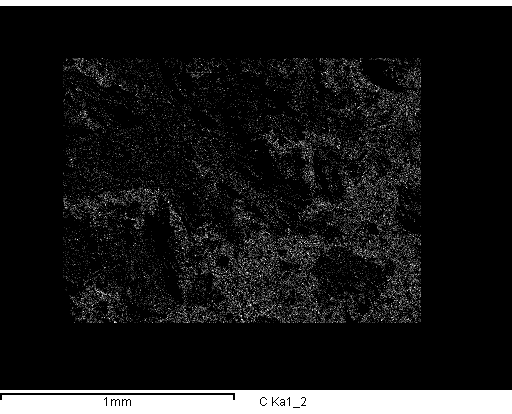 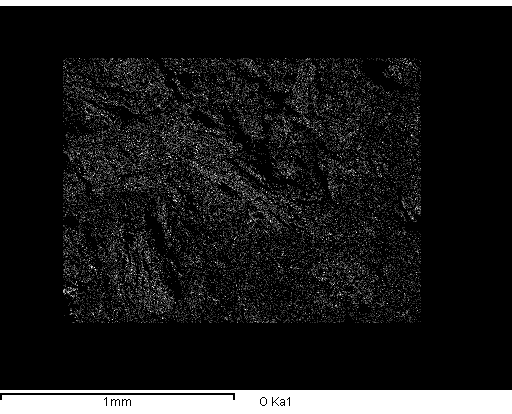  Na Mg  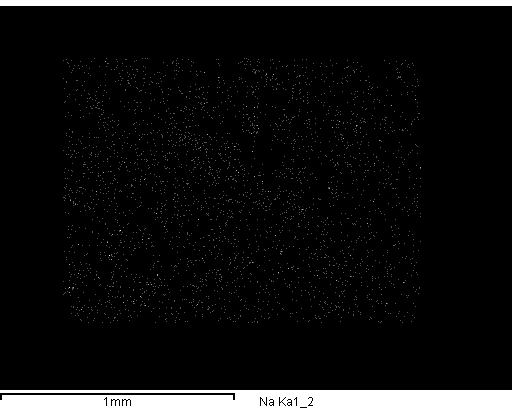 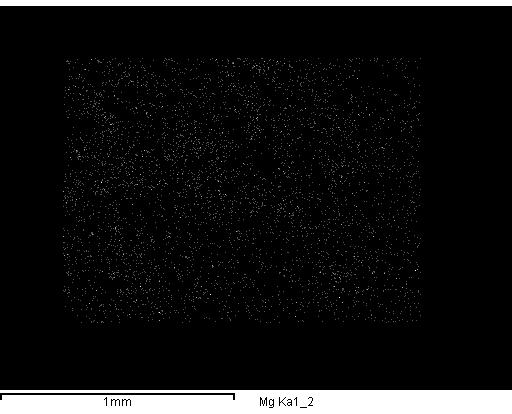  Al  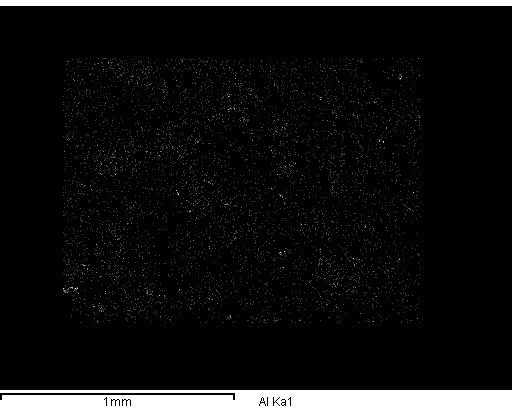 |
| 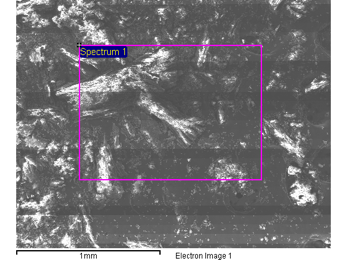 | 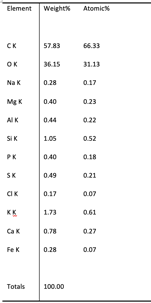 | 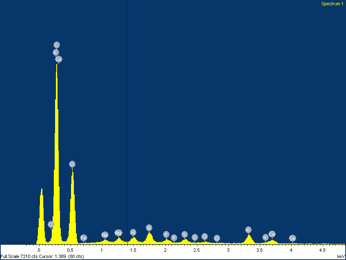 |  |
| 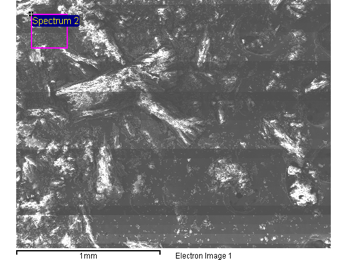 | 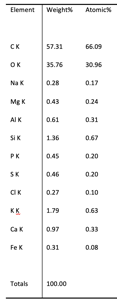 | 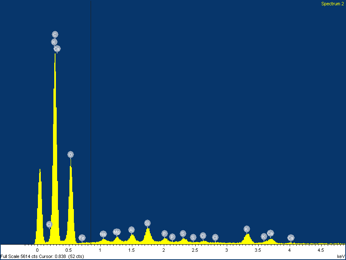 |  |
| 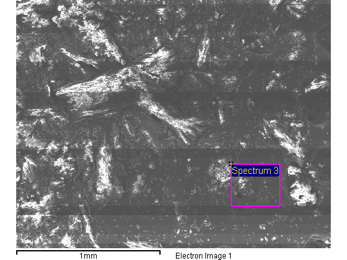 | 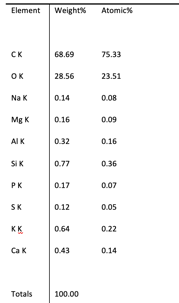 | 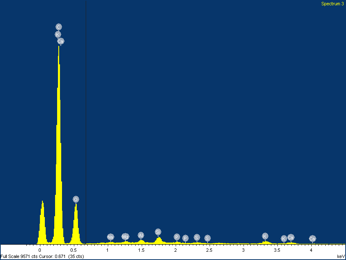 |  |
| 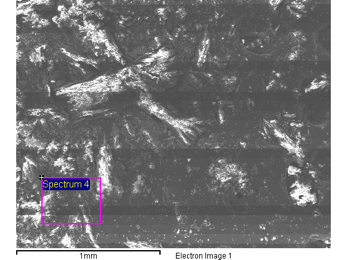 | 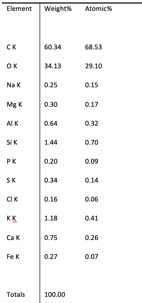 | 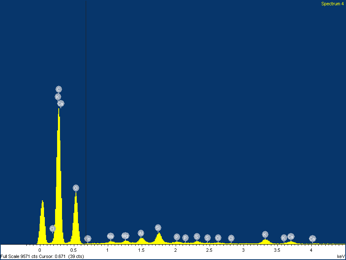 |  |

- 1. Element composition data and mapping image for shoot system of sweet corn.

| SEM | EDX data | EDX spectrum | Mapping image | | |
| --- | --- | --- | --- | --- | --- |
| 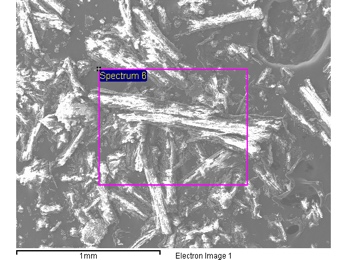 | 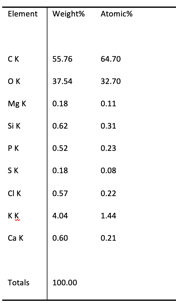 | 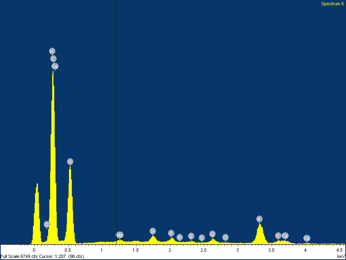 | C  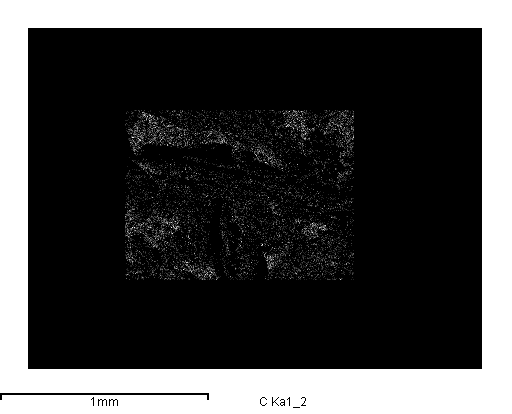 | O  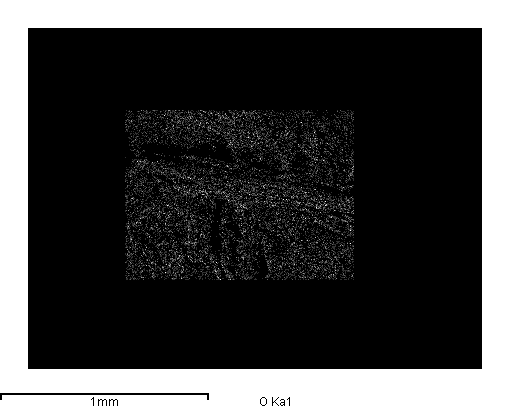 | Na  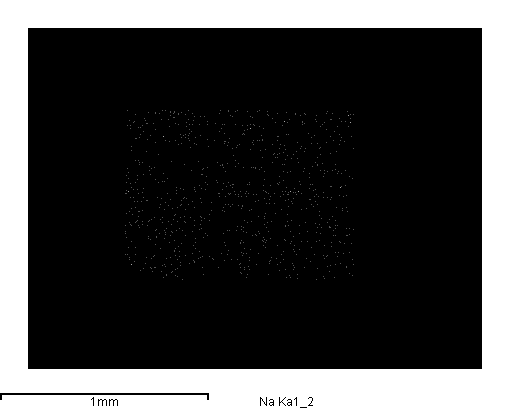 |
| 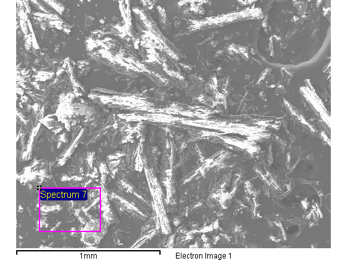 | 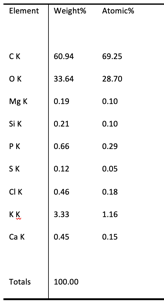 | 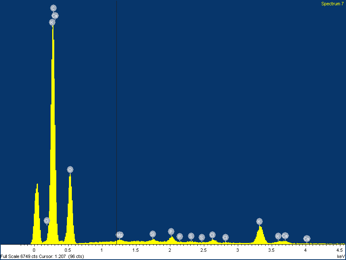 | Mg  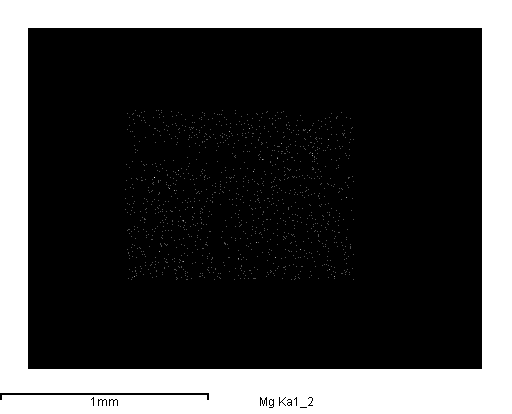 | Al  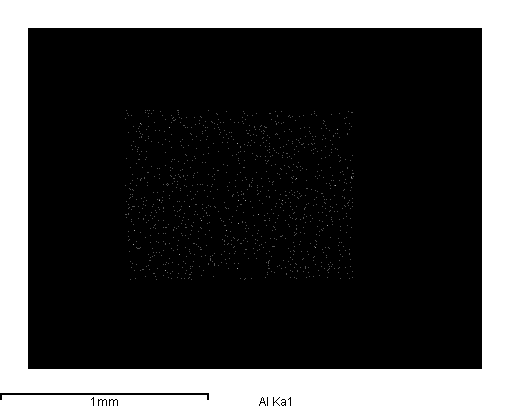 | Si  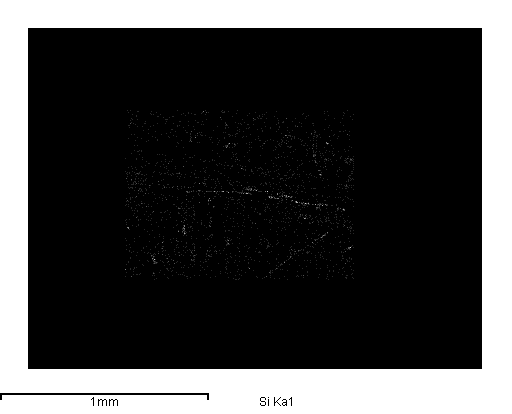 |
| 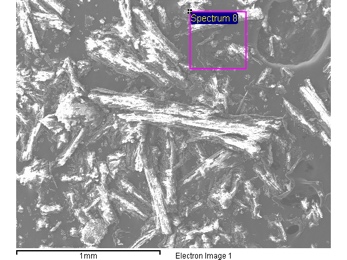 | 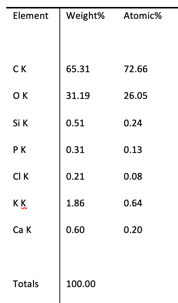 | 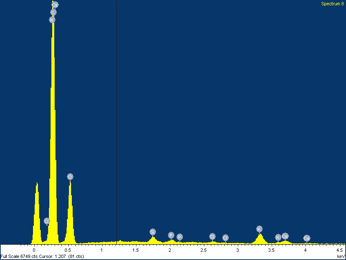 | P  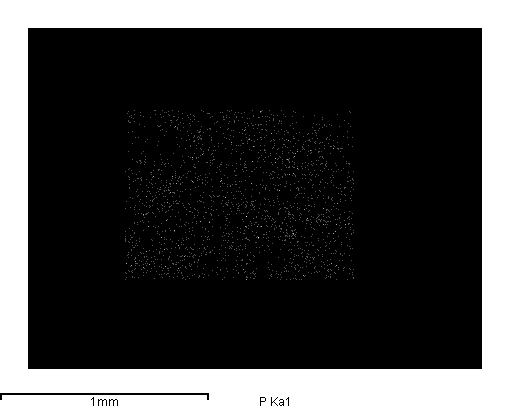 | S  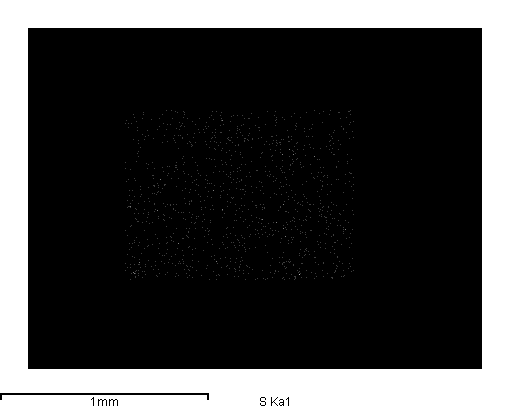 | Cl  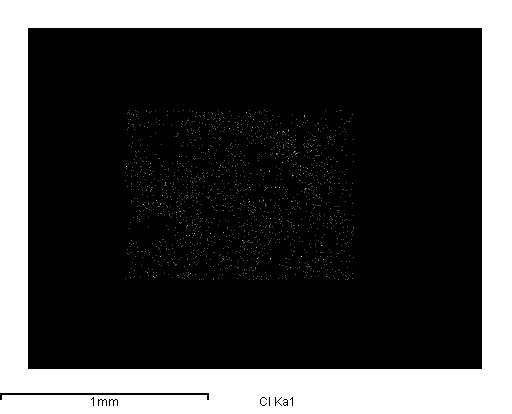 |
| 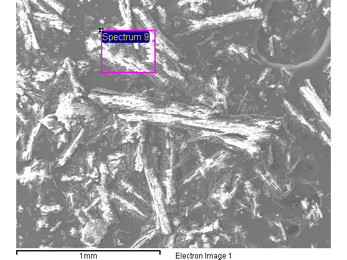 | 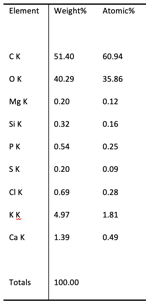 | 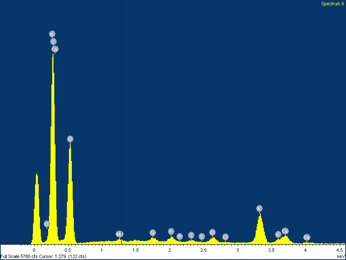 | K  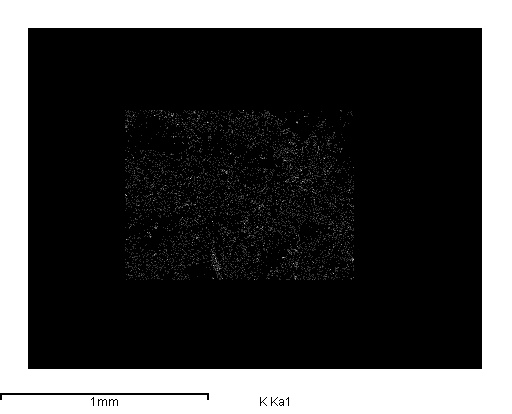 | Ca  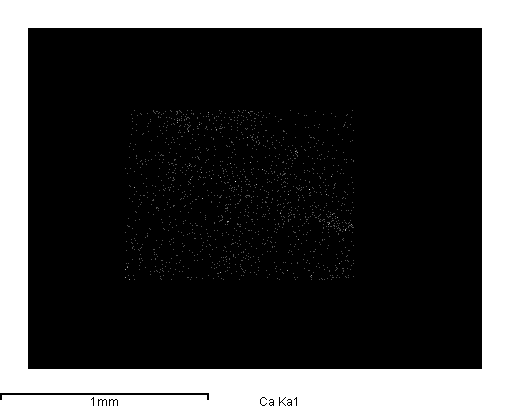 | Fe  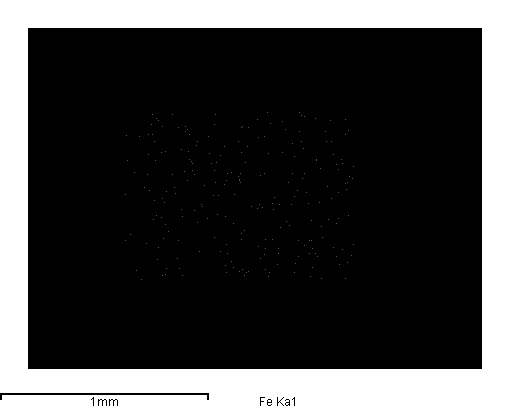 |

1. ***Cu_2_(OH)(BTC)(H_2_O)]_n_·2nH_2_O***
   1. Element composition data and mapping image for the root of sweet corn.

| SEM | EDX data | EDX spectrum | Mapping image | | |
| --- | --- | --- | --- | --- | --- |
| 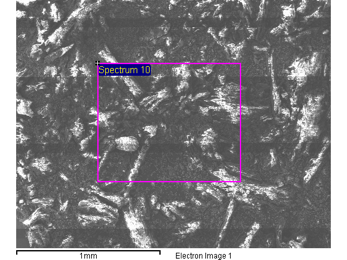 | 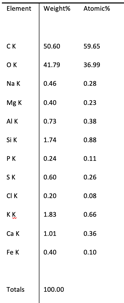 | 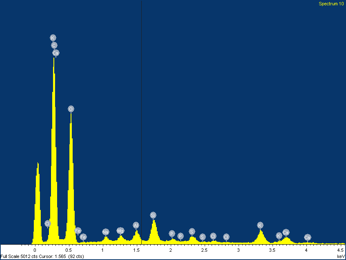 | C  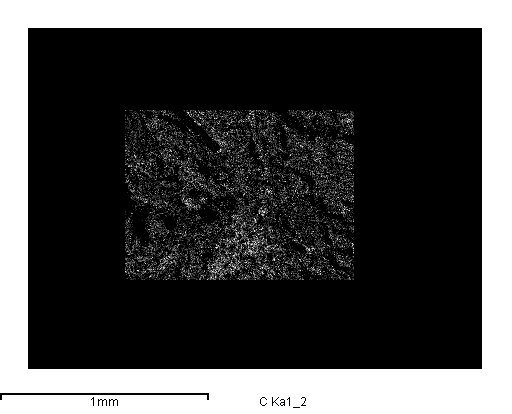 | O  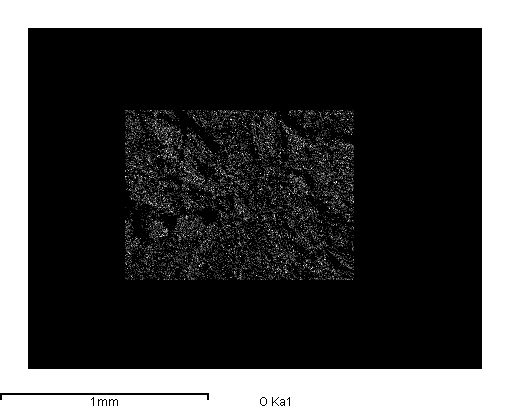 | Na  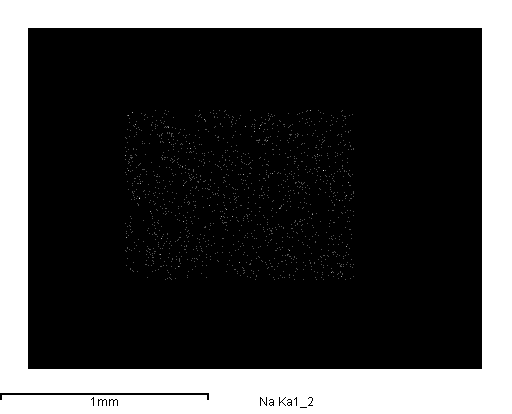 |
| 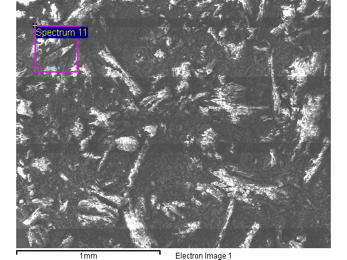 | 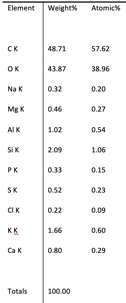 | 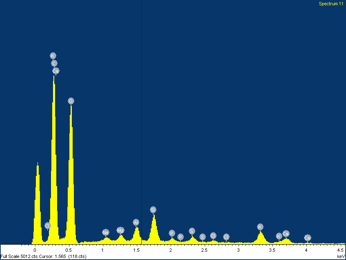 | Mg  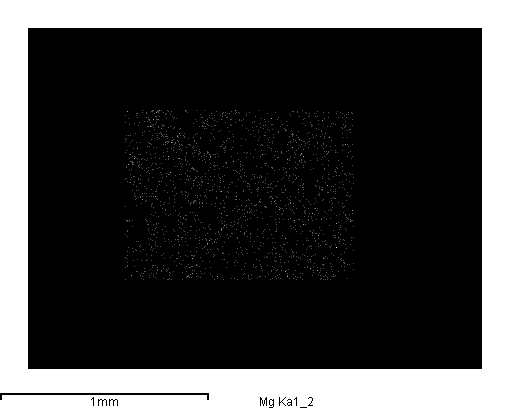 | Al  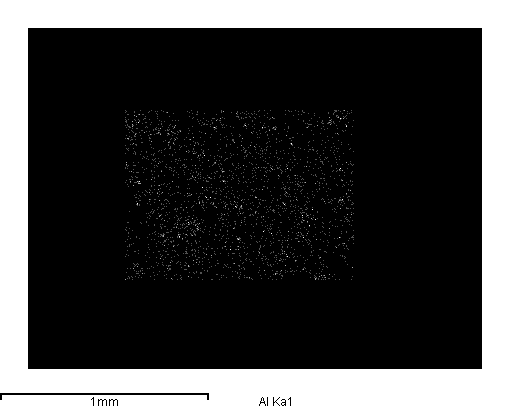 | P  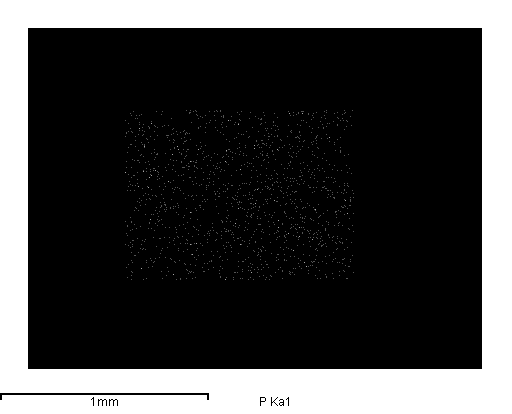 |
| 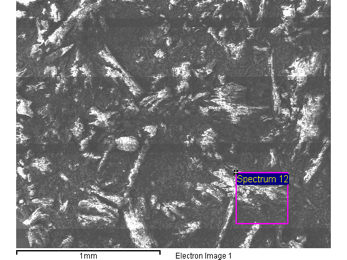 | 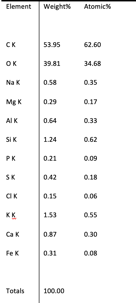 | 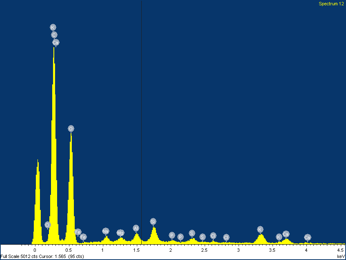 | S  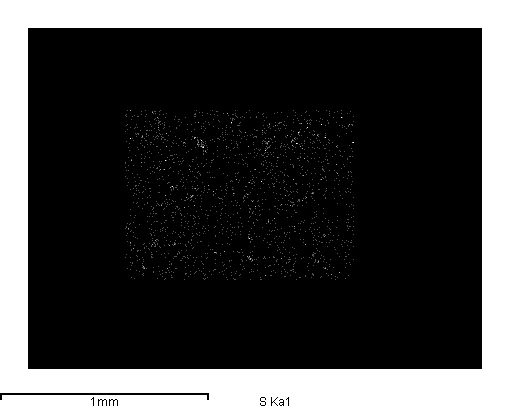 | Cl  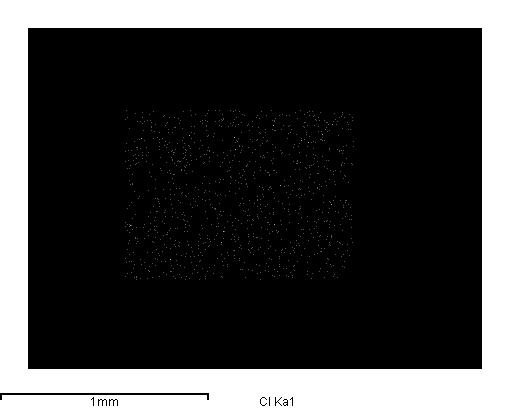 | K  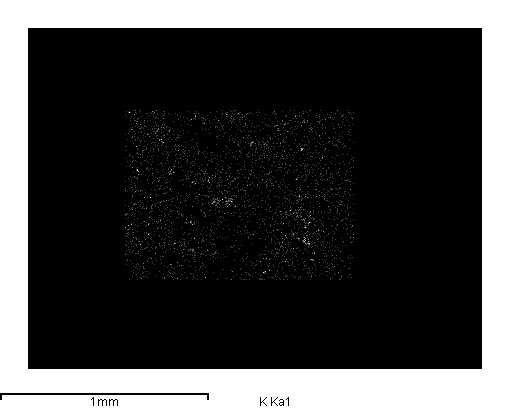 |
| 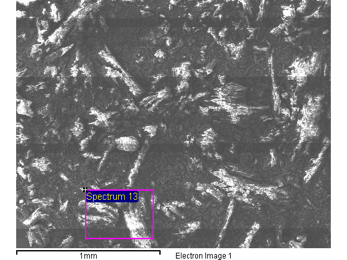 | 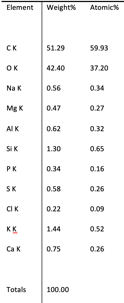 | 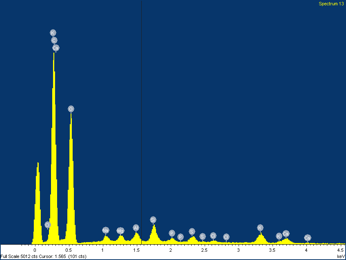 | Ca  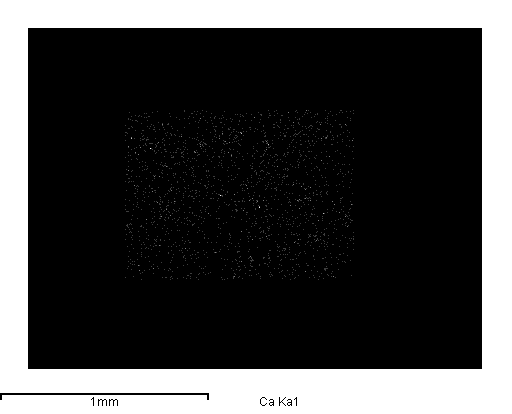 | | |

- 1. Element composition data and mapping image for shoot system of sweet corn.

| SEM | EDX data | EDX spectrum | Mapping image | | |
| --- | --- | --- | --- | --- | --- |
| 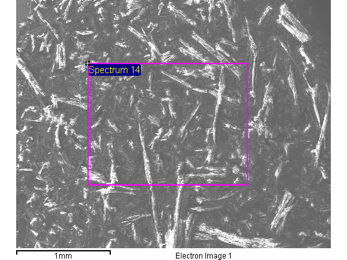 | 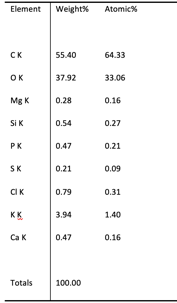 | 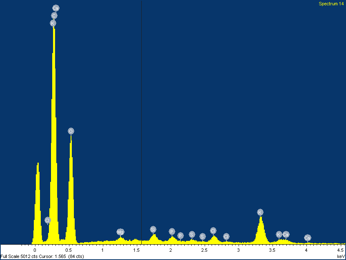 | C  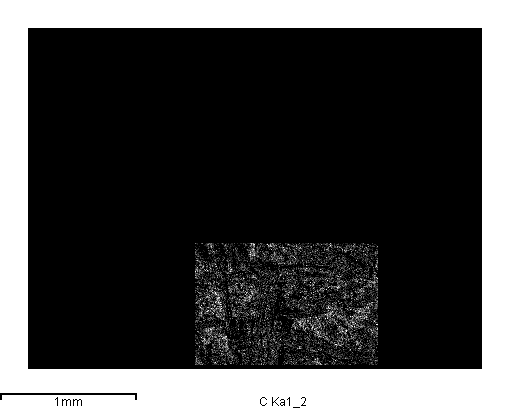 | O  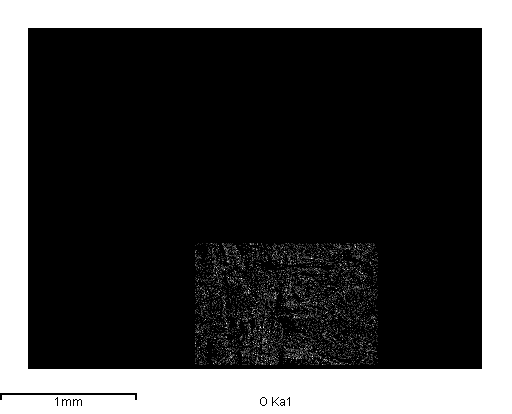 | Mg  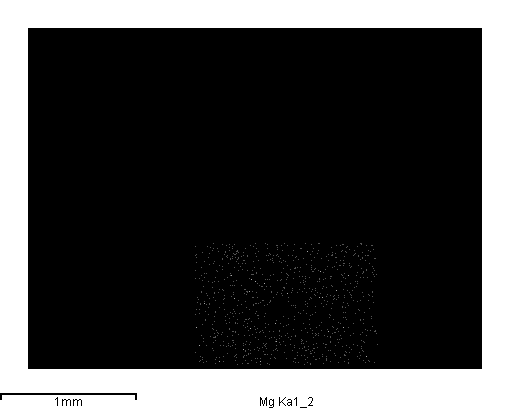 |
| 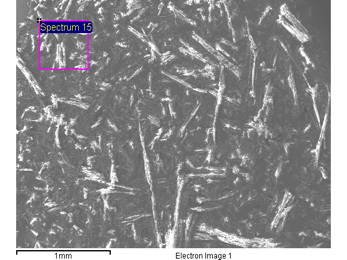 | 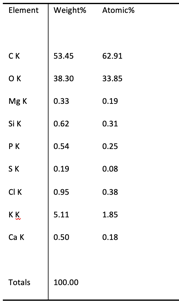 | 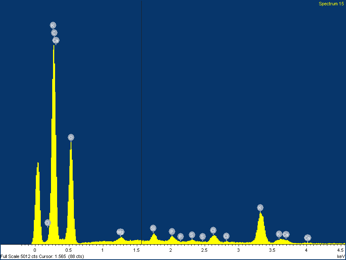 | Si  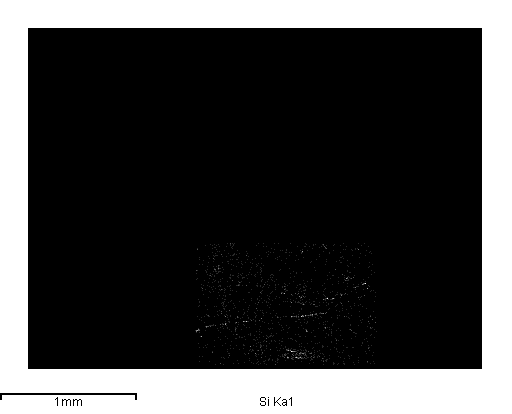 | K  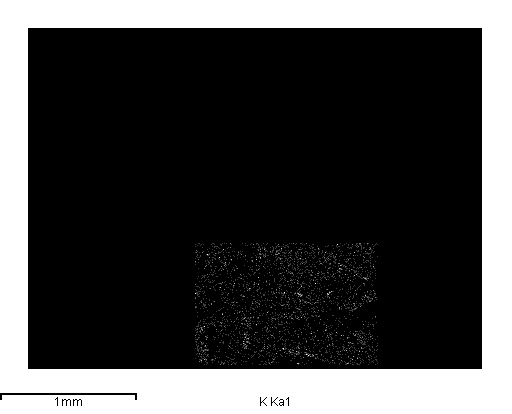 | Ca  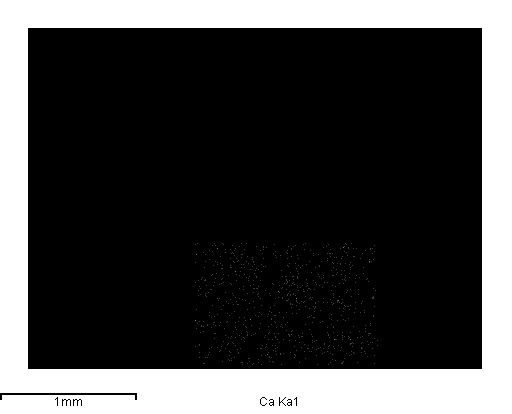 |
| 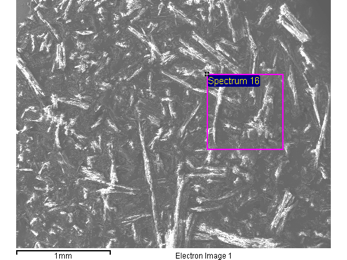 | 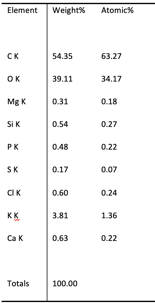 | 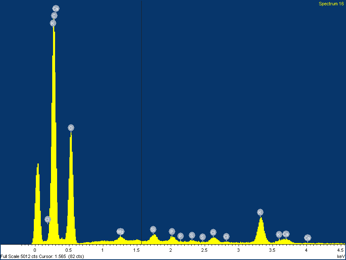 | P  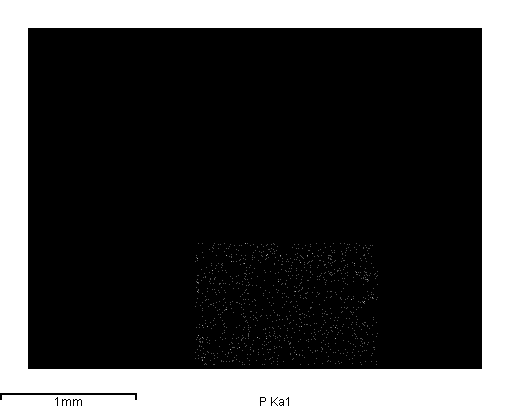 | S  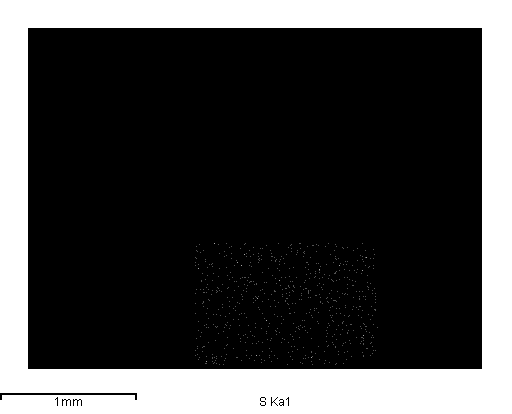 | Cl  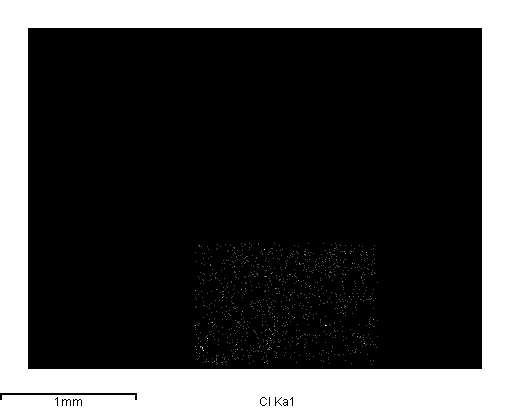 |

1. ***HKUST-1***
   1. Element composition data and mapping image for the root of sweet corn.

| SEM | EDX data | EDX spectrum | Mapping image |
| --- | --- | --- | --- |
| 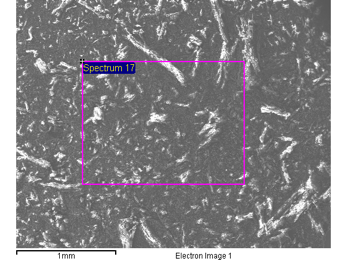 | 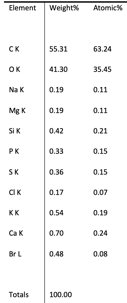 | 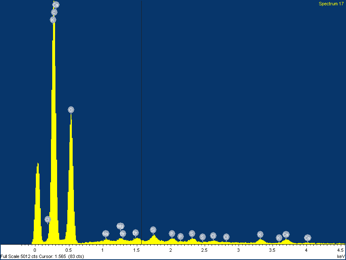 | C O Na  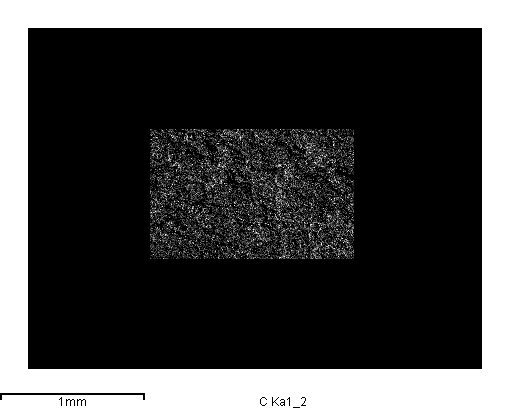 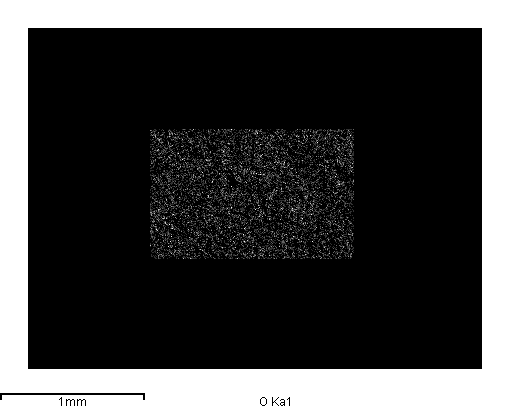 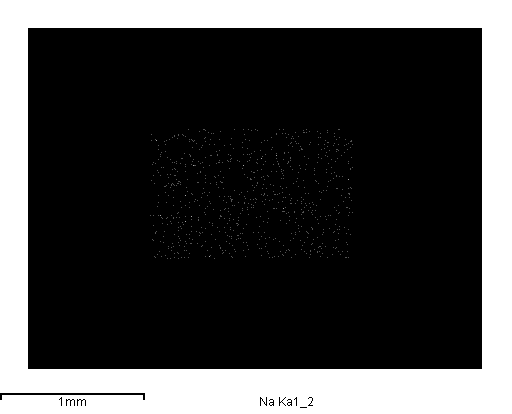  Mg Al Si  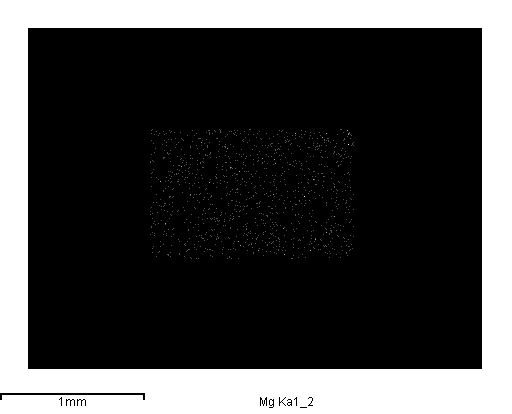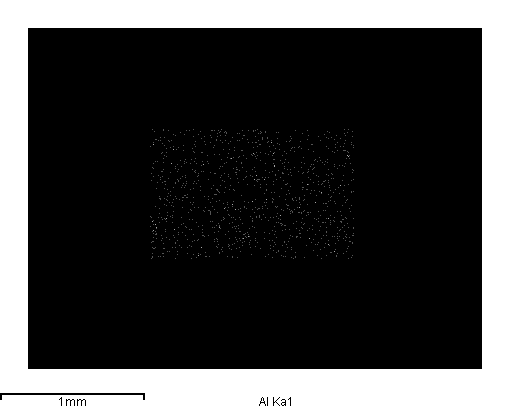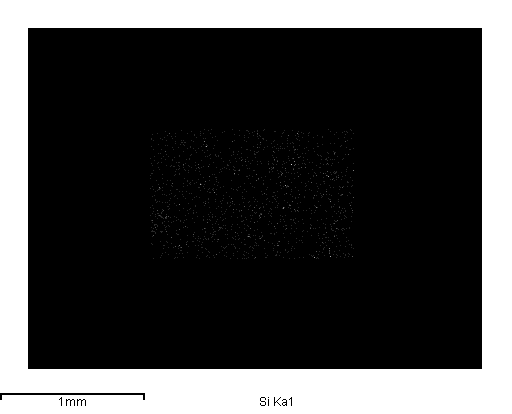  P S Cl  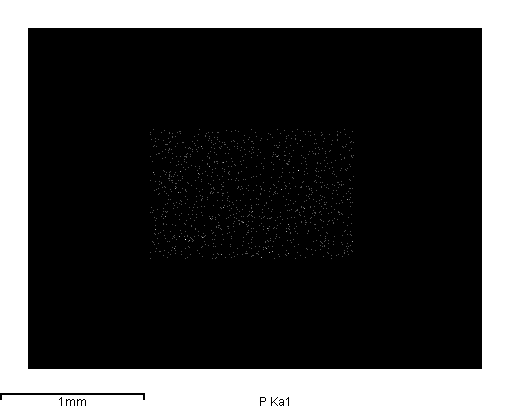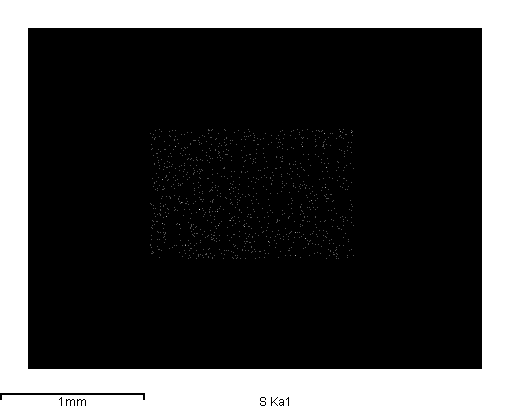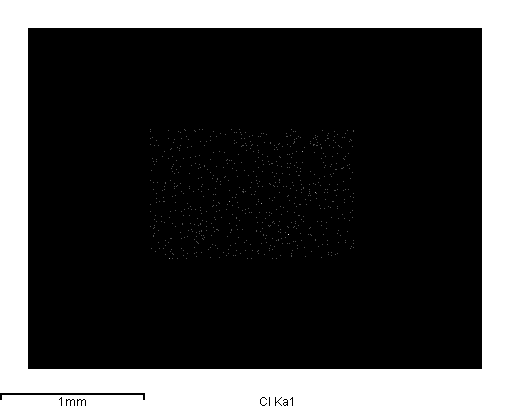  K Ca  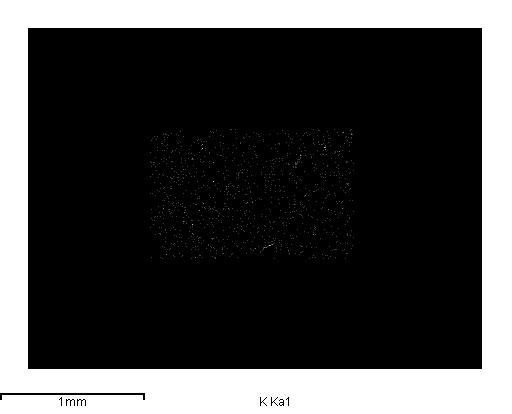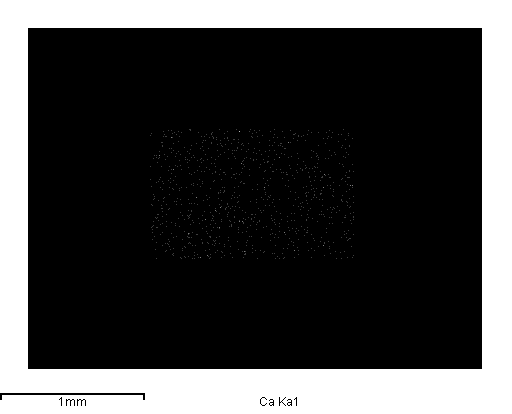 |
| 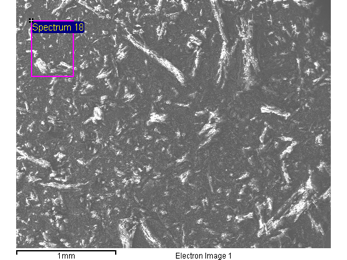 | 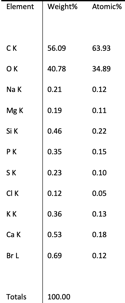 |  |  |
|  |  |  |  |

- 1. Element composition data and mapping image for shoot system of sweet corn.

| SEM | EDX data | EDX spectrum | Mapping image | | |
| --- | --- | --- | --- | --- | --- |
|  |  |  | C | O | Si |
|  |  |  | Mg | S | Cl |
|  |  |  | P | Ca | K |
